# Immediate Early Response Gene X-1 (IEX-1) Mediates Ischemic Preconditioning-Induced Cardioprotection in Rats

**DOI:** 10.1155/2017/6109061

**Published:** 2017-10-29

**Authors:** Ming-Jiang Xu, Yan Cai, Aijuan Qu, John Y.-J. Shyy, Wenjing Li, Xian Wang

**Affiliations:** ^1^Department of Physiology and Pathophysiology, School of Basic Medical Science, Peking University Health Science Center, Key Laboratory of Molecular Cardiovascular Science, Ministry of Education, Beijing, China; ^2^Basic Medical School, Wuhan University, Wuhan, China; ^3^Department of Physiology and Pathophysiology, School of Basic Medical Science, Capital Medical University, Key Laboratory of Remodeling-Related Cardiovascular Diseases, Ministry of Education, Beijing, China; ^4^Department of Medicine, University of California, San Diego, San Diego, CA 92093, USA

## Abstract

Reversible myocardial ischemia/reperfusion (I/R) or ischemic preconditioning (IPC) is associated with an immediate genomic response; IPC-induced immediate early genes are associated with reduced infarct size. Because the immediate early response gene X-1 (IEX-1) plays a central role in cell apoptosis, we examine whether IEX-1 exerts protective effects against I/R injury. We found that the IEX-1 mRNA level was increased in the IPC-imposed rat heart. However, it was downregulated in the I/R rat heart, which was prevented by in situ IPC. When IEX-1 was knocked down, the protective effects imposed by IPC were lessened. Local gene delivery of Ad-IEX-1 to the left ventricle greatly diminished cardiac infarct size and improved systolic functions of I/R hearts in rats. In contrast, knocking down IEX-1 expression exacerbates myocardial infarction. Overexpression of IEX-1 in neonatal rat cardiomyocytes significantly reduced hypoxia-reoxygenation-induced intracellular and mitochondrial ROS accumulation and cell apoptosis. Furthermore, IPC-induced phosphorylation and particle translocation of PKCε were impaired by knocking down IEX-1 in vivo, and overexpressing IEX-1 showed similar cardioprotection imposed by IPC. Our results demonstrate that IPC increases IEX-1 expression, which may promote phosphorylation and particle translocation of PKCε and thus reduce intracellular ROS accumulation. These beneficial effects reduce cardiomyocyte apoptosis and necrosis to alleviate cardiac infarction.

## 1. Introduction

Timely reperfusion after myocardial ischemia is the definitive strategy to salvage myocardium at risk of lethal injury. However, abrupt restoration of blood flow to the ischemic myocardium carries the potential of introducing additional cardiomyocyte injury and death [1, 2]. Such reperfusion injury (RI) involves opening of the mitochondrial permeability transition pore (MPTP) under the conditions of calcium overload and oxidative stress that accompany reperfusion. Protection from MPTP opening and hence RI can be mediated by ischemic preconditioning (IPC) where the prolonged ischemic period is preceded by one or more brief cycles of ischemia and reperfusion [3, 4].

There is a plethora of data implicating many different signaling pathways in preconditioning, and the relevant role of each remains hotly debated. And of them, there is extensive evidence that protein kinase C (PKC) plays a central role in preconditioning [5]. There remains some controversy over which of the many PKC isoforms may be involved in IPC. Nevertheless, there is a large body of evidence to implicate PKC*ε* and as important players in IPC [5]. The proposed mechanism of PKC*ε*-mediated cardioprotection is that generation of mitochondrial ROS during IPC may lead to activation of PKC*δ*. This increases adenosine generation and release leading to activation of phospholipase C. Generation of diacylglycerol and inositol-triphosphate results in calcium release and activation of PKC*ε* [5]. However, there is rare study about whether PKC activation is regulated by immediate early genes.

IPC, that is, cyclic episodes of short durations of ischemia and reperfusion (I/R), potentiates redox signaling to convert the death signals into survival ones to protect the ischemic heart [6]. A previous study has shown that IPC induces the expression of proto-oncogenes or immediate early genes such as c-Fos, c-Myc, c-Jun, and Egr-1 in isolated rat hearts, which was associated with improved ventricular function and reduced infarct size [7–9], but the underlying mechanism involving in immediate early genes protecting an ischemia heart remains elusive.

Immediate early response gene X-1 (IEX-1), also known as p22/PRG1 in rat or the mouse homologue gly96, is a novel stress-induced immediate early gene. It can be rapidly upregulated in various cell types by irradiation, viral infection, inflammatory cytokines, chemical carcinogens, growth factors, and hormones under the control of transcription factors such as NF-*κ*B/rel, p53, Sp1, c-Myc, and AP-1 (reviewed by Wu) [10]. Like other immediate early genes, IEX-1 plays a pivotal role in cell survival under stress conditions [11–16]. Decreased IEX-1 expression is proved to associate with enhanced apoptosis in a titin-deficient mouse model of dilated cardiomyopathy [17]. Furthermore, IEX-1 has the ability of regulating several protein kinase activities, such as ERK and Akt, through interacting with protein phosphatase 2A (PP2A) [18, 19].

In the present study, we examine whether IEX-1 exerts protective effects against I/R injury imposed on rat neonatal and adult hearts. We found that IEX-1 expression was downregulated in the ischemic rat heart, but was rescued by IPC. The restored IEX-1 expression not only decreased the I/R-induced apoptosis and necrosis but also alleviates cardiac infarction. The putative mechanisms include increased activation of PKC and attenuated I/R-induced ROS accumulation.

## 2. Materials and Methods

### 2.1. Adenoviral Vector Construction

Replication-deficient adenoviral vectors containing human IEX-1 cDNA (Ad-IEX-1) or GFP (Ad-GFP) under the control of the cytomegalovirus (CMV) promoter were constructed and prepared according to the manufacturer's instruction (BD Biosciences, San Jose, CA). Adenoviral vectors carrying bacterial *β*-galactosidase (Ad-*β*-Gal) were kindly provided by Prof. H. Cheng (Peking University, China).

### 2.2. Rat Model of Myocardial Ischemia and IPC

All animals received humane care in compliance with the Institutional Authority for Laboratory Animal Care of Peking University Health Science Center. The Sprague-Dawley (SD) rat heart ischemia and IPC surgical procedures were described previously [20]. Briefly, after thoracotomy, rats underwent 30 min of myocardial ischemia, then 3 or 24 h of reperfusion. For IPC treatment, before lethal I/R, rats underwent three cycles of short I/R (4 min/4 min). Local gene delivery was performed with protocols previously described [21]. Four days before ischemia, 5 × 10^9^ plaque-forming unit (pfu) adenovirus in 250 *μ*l of virus solution was injected intramyocardially into the left ventricular muscular wall.

### 2.3. *In Vivo* Small Interfering RNA Repression

We injected siRNA/transfection agent solution into the left ventricle muscle wall to achieve sufficient siRNA concentrations for IEX-1 repression within cardiac tissues. Rats were anesthetized by intraperitoneal injection of sodium pentobarbital (60 mg/kg). After thoracotomy, siRNA (20 *μ*g siRNA/kg body weight) together with transfection agent solution (jetPEI, PolyPlus transfection) was intramyocardially injected in 5 predetermined regions in the left anterior descending (LAD) area. A total of 250 *μ*l of siRNA/transfection agent solution was delivered. Injections were performed by a single investigator in a blinded fashion. Stealth siRNA targeting rat IEX-1 (sense: cca aca uug cca aga gga ucc ucu u; antisense: aag agg auc cuc uug gca aug uug g) and control siRNA (Cat number: 12935300) were synthesized by Invitrogen (Invitrogen, CA). Two days after injection, cardiac tissue around the injected area was excised, and IEX-1 levels were determined by real-time PCR and Western blot.

### 2.4. Neonatal Rat Ventricular Cardiomyocyte Culture

Neonatal rat ventricular cardiomyocytes were isolated from 1- to 2-day-old Sprague-Dawley rats [22]. Experiments were performed at day 3 in culture. For hypoxia/ reoxygenation (H/R), cells were cultured in hypoxia buffer (in mmol/l NaCl 118, NaHCO_3_ 24, KCl 16, KH_2_PO_4_ 1, CaCl_2_ 2.5, MgCl_2_ 1.2, Na^+^ EDTA 0.5, and Na^+^ lactate 20, pH 6.2) and underwent <1% O_2_ + 5% CO_2_ + argon treatment for 4 h, then were transferred to 5% CO_2_ + 95% air for reoxygenation. And 10 min hypoxia followed with 10 min reoxygenation was considered as one cycle of preconditioning.

### 2.5. Real-Time PCR and Western Blot Analysis and Immunostaining

For details of real-time PCR, Western blot analysis, and immunostaining, see the online-only Data Supplement available online at https://doi.org/10.1155/2017/6109061.

### 2.6. Measurement of ROS Accumulation

The accumulation of intracellular ROS and mitochondrial ROS was detected with luminol-derived chemiluminescence, DCFH-DA, and MitoTracker® Red CM-H2XRos (for details, see the online-only Data Supplement).

### 2.7. Statistical Analysis

All values are expressed as mean ± SEM. Comparisons between more than 2 groups were analyzed by one-way ANOVA followed by Tukey-Kramer post hoc testing. A *P* < 0.05 was considered statistically significant.

The authors had full access to the data and take responsibility for its integrity. All authors have read and agree to the manuscript as written.

## 3. Results

### 3.1. IEX-1-Mediated IPC-Induced Cardioprotection

**Figure 1:**
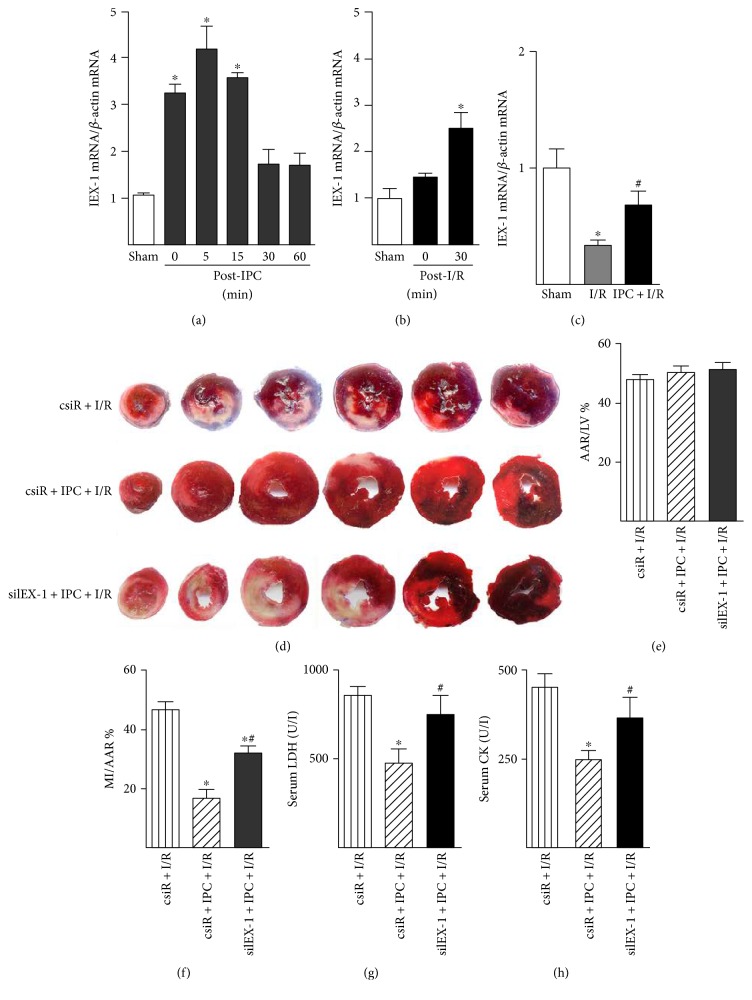
IEX-1-mediated IPC-induced cardioprotection. (a) IEX-1 mRNA level in rat hearts post-IPC was detected by quantitative real-time PCR (*n* = 5~6). (b) IEX-1 mRNA level in rat hearts postischemia (30 min) or I/R (30 min/30 min). (c) IEX-1 mRNA level in rat hearts subjected to sham operation (Sham), I/R (30 min/3 h), or IPC prior I/R (*n* = 6~10). (d). 5 *μ*g scrambled siRNA (csiR) or siRNA targeting IEX-1 (siIEX-1) with transfection reagent in 250 *μ*l of saline was directly injected intramyocardially into the left ventricular muscular wall. 2 days after the injection, rats underwent I/R (30 min/24 h) or IPC prior I/R (*n* = 6~7), then hearts were excised and sliced for infarction analysis by Evans blue and triphenyltetrazolium chloride (TTC) attaining; (e) percentage of the area at risk (AAR) to the left ventricle (LV) and (f) infarct area (MI) to AAR were determined by computer-assisted planimetry. (g) Serum lactate dehydrogenase (LDH) and (h) creatine kinase (CK) activity were measured at the end of 24-hour reperfusion. *n* = 7~10, ^∗^*P* < 0.05 versus csiR + I/R, ^#^*P* < 0.05 versus csiR + IPC + I/R.

We used a model of in situ IPC of intermittent coronary occlusion to examine first the expression of IEX-1 in the rat heart. As shown in Figure 1(a), the IEX-1 mRNA level was increased rapidly in the IPC-imposed heart, peaked at 5 min, and declined thereafter, which remained higher than the basal level at 1 h (Figure 1(a)). Lethal ischemia (30 min) itself does not promote IEX-1 expression, although the IEX-1 mRNA level increased at early reperfusion (30 min) (Figure 1(b)); it decreased at 3 h reperfusion (Figure 1(c)), and the level of IEX-1 mRNA was higher in hearts that underwent IPC prior to I/R than that in hearts receiving I/R only (Figure 1(c)). The IEX-1 protein level decreased at 3 h reperfusion which was rescued by IPC prior to I/R (Supplemental Figure 1A and B).

Because IPC could induce IEX-1 expression, we then investigated whether the increased level of IEX-1 is involved in IPC-mediated cardiac protection. Small interfering RNA targeting rat IEX-1 (siIEX-1) was used to knock down IEX-1 expression in hearts. As shown in Figures 1(d), 1(e), and 1(f), in situ IPC protected the heart, revealed by decreased infarct size, in the scrambled siRNA (csiR) group, but not in the siIEX-1 group. These were parallel, confirmed by decreased release of lactate dehydrogenase (LDH) and creatine kinase (CK) to serum in the csiR group, but not in the siIEX-1 group (Figures 1(g) and 1(h)).

**Figure 2:**
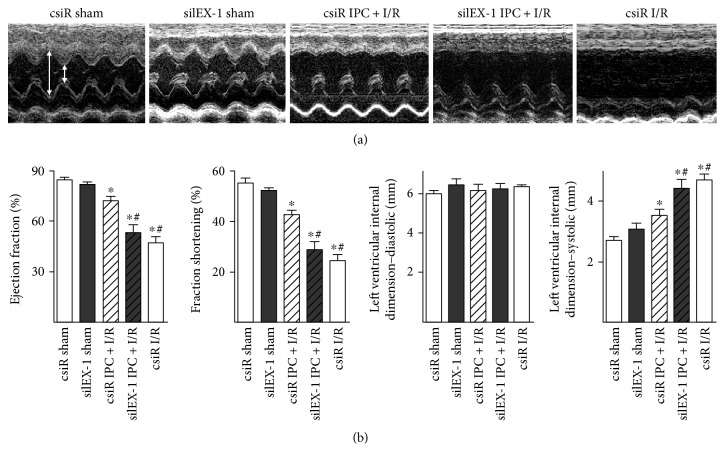
Echocardiography-assessed cardiac function. (a) Echocardiographic images showing systolic and diastolic dimensions (arrows) measured by M-mode in the parasternal short axis view at the level of the papillary muscles. (b) Summarized mean echo data of ejection fraction, fractional shortening, diastolic internal dimension, and systolic internal dimension. *n* = 9~14, ^∗^*P* < 0.05 versus csiR sham, ^#^*P* < 0.05 versus csiR IPC + I/R.

IEX-1 knockdown impaired the protective effects of IPC, which was further confirmed by echocardiography analysis. As shown in Figures 2(a) and 2(b), impaired systolic function post-I/R was observed in the I/R group, assessed by ejection fraction (EF), fraction shortening (FS), and systolic left ventricular internal dimension (LVID-s), while IPC-imposed rats showed improved systolic function post-I/R in the csiR group. Of note, depraved systolic function was observed in the rats treated with siIEX-1 prior to IPC, which was similar to that in I/R alone rats.

### 3.2. IEX-1 Overexpression Attenuates Myocardial Infarction

**Figure 3:**
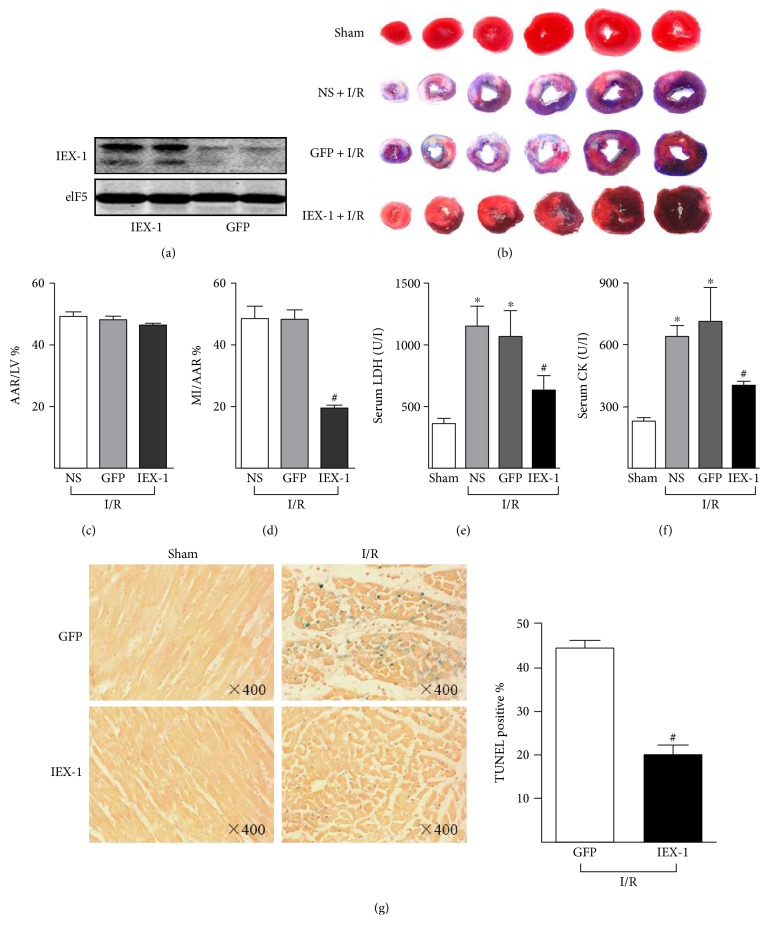
IEX-1 overexpression attenuates myocardial infarction. Rats were injected with saline (NS) (*n* = 7), Ad-GFP (*n* = 11), or Ad-IEX-1 (*n* = 8) for 4 days, then underwent I/R (30 min/24 h). (a) Western blot analysis confirmed overexpression of IEX-1 in the left ventricle after four days of local adenovirus delivery. (b) Representative images of the infarct heart stained by Evans blue and TTC. (c) Ratios of AAR to LV and (d) MI to AAR were shown. (e) Serum LDH and (f) CK activity were measured at the end of 24-hour reperfusion. (g) After subjected to I/R, hearts were excised for slicing, then TUNEL staining was performed for assessing cardiac cell apoptosis post-I/R. ^∗^*P* < 0.05 versus Sham, ^#^*P* < 0.05 versus GFP.

To investigate a functional contribution of IEX-1 to postischemic cardioprotection, we developed an adenoviral vector expressing human IEX-1 (Ad-IEX-1). In the experimental group, Ad-IEX-1 was directly injected into the left ventricle wall, whereas control groups received Ad-GFP or saline. Four days post the injection, rat hearts were excised. The overexpression of IEX-1 in the cardiac muscle was confirmed by Western blot (Figure 3(a)) and immunostaining (Supplemental Figure 2A). As shown in Figures 3(b), 3(c), and 3(d), IEX-1 overexpression caused significant cardioprotection. A significant decrease in infarct size of the hearts was found in these animals, when compared with that of hearts receiving saline or Ad-GFP. In addition, these rats exhibited lower levels of serum LDH and CK (Figures 3(e) and 3(f)). As well, the accident rate of cell apoptosis in the area at risk, assessed by TUNEL staining, was significantly reduced in IEX-1 overexpressed rats (Figure 3(g)).

**Figure 4:**
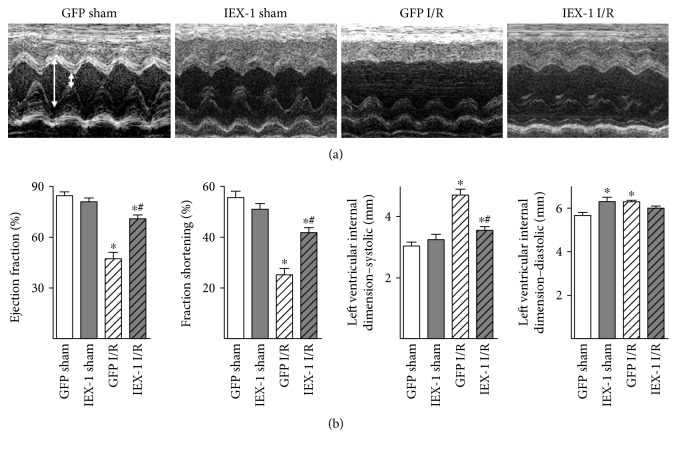
IEX-1 overexpression improved cardiac function post-I/R assessed by echocardiography. (a) Echocardiographic images showing systolic and diastolic dimensions (arrows) measured by M-mode in the parasternal short axis view at the level of the papillary muscles. (b) Echo data of ejection fraction, fractional shortening, systolic internal dimension, and diastolic internal dimension. *n* = 7~9, ^∗^*P* < 0.05 versus GFP sham, ^#^*P* < 0.05 versus GFP I/R.

To examine the functional consequences resulting from Ad-IEX-1 gene delivery, we measured cardiac systolic function by echocardiography and measurement of cardiac hemodynamics parameters. Of note, impaired systolic functions post-I/R, assessed by EF, FS, and LVID-s, were observed in the GFP group, while Ad-IEX-1 delivery rats showed improved post-I/R systolic functions (Figures 4(a) and 4(b)). Similarly, catheter-mediated measurement of cardiac hemodynamics parameters showed that mean arterial pressure, left ventricular systolic pressure, maximal rates of pressure increase and decrease were significantly impaired post-I/R, whereas these parameters were improved in Ad-IEX-1 delivery rats (Supplemental Table 1).

### 3.3. SiRNA Knocking Down IEX-1 Exacerbates Myocardial Infarction

**Figure 5:**
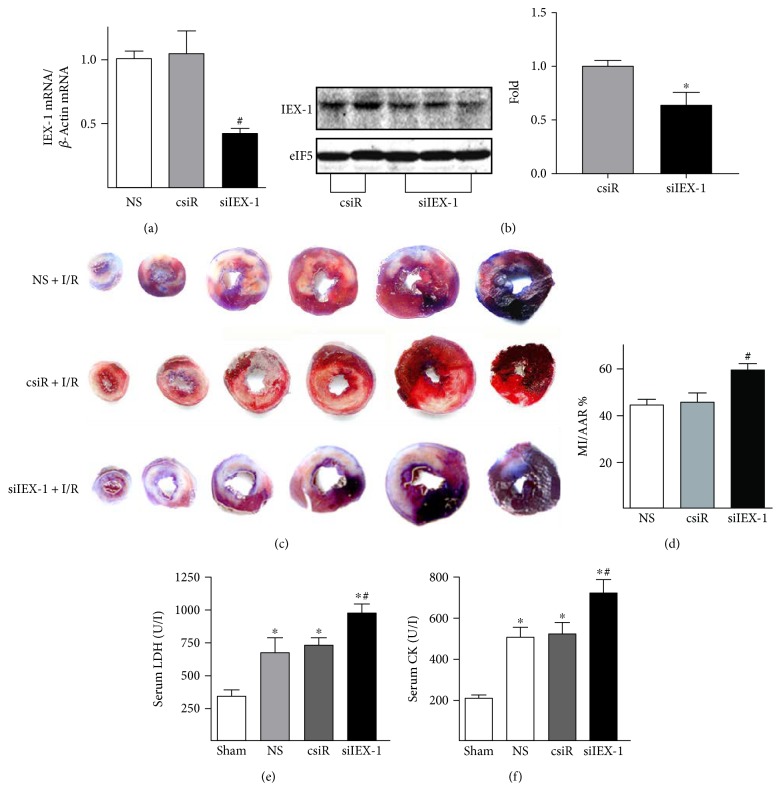
siRNA repression of IEX-1 exacerbates myocardial infarction. 5 *μ*g csiR or siIEX-1 with transfection reagent in 250 *μ*l of saline (NS) was directly injected intramyocardially into the left ventricular muscular wall. 2 days after, (a) IEX-1 mRNA levels and (b) protein levels were determined. (c) 2 days after siRNA injection, rats underwent I/R (30 min/24 h). Representative images of heart infarction assessed by Evans blue and TTC staining were shown. (d) Infarct sizes are expressed as ratio of MI to AAR. (e) Serum LDH and (f) CK activity were measured at the end of 24 h reperfusion. *n* = 5 in each group. ^∗^*P* < 0.05 versus sham, ^#^*P* < 0.05 versus NS or csiR.

In the reciprocal approach, we used siRNA to repress the cardiac expression of IEX-1 in vivo. An amount of 5 *μ*g of IEX-1 siRNA (siIEX-1) together with transfection reagent was directly injected into the rat left ventricular wall. The IEX-1 mRNA level was reduced at 48 h postinjection (Figure 5(a)), and the corresponding reduction of protein was confirmed by Western blot (Figure 5(b)) and immunostaining (Supplemental Figure 2B). Responding to I/R (30 min/24 h), rats with siIEX-1 injection showed significantly increased infarct size expressed as the percentage of the left ventricle area (~7%), when compared with animals with control siRNA (Figures 5(c) and 5(d)). Similarly, plasma LDH and CK activity were increased in rats receiving siIEX-1 (Figure 5(e) and 5(f)).

### 3.4. IEX-1 Inhibits H/R-Induced ROS Accumulation

**Figure 6:**
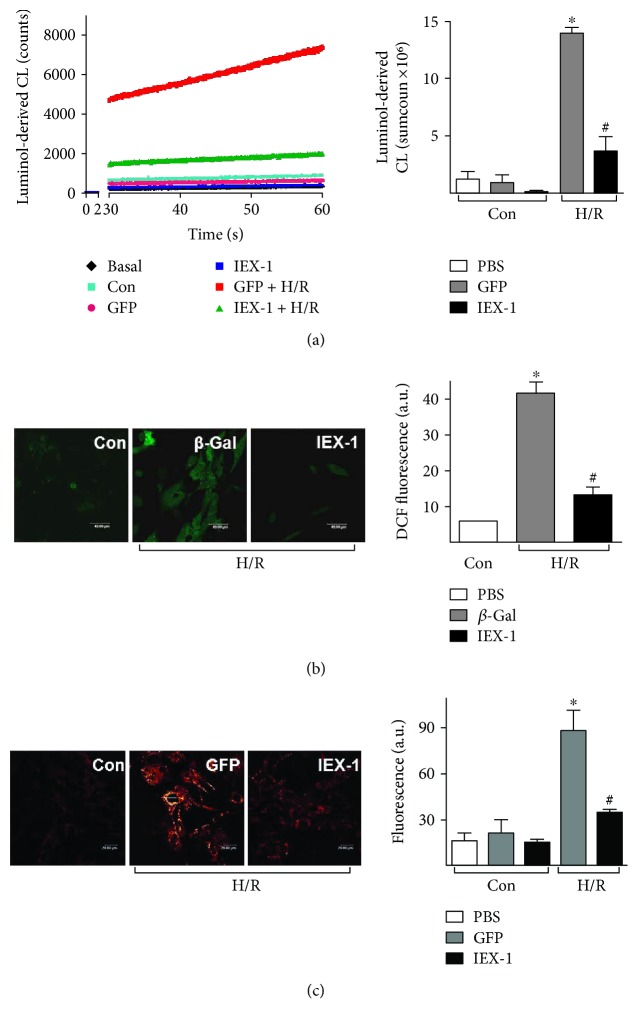
IEX-1 overexpression inhibits H/R-induced ROS accumulation. Neonatal rat cardiomyocytes were infected with adenovirus at 10 MOI for 36 h, then underwent H/R (4 h/30 min). (a) ROS levels measured by luminol chemiluminescence (left). The column data are the sum counts of the 30-minute measurement period (right). (b) Intracellular ROS were detected by DCFH-DA (scale bar = 40 *μ*m), and (c) mitochondrial ROS were detected by MitoTracker Red CM-H2XRos probe with confocal microscopy (scale bar = 20 *μ*m). ^∗^*P* < 0.05 versus Ctrl, ^#^*P* < 0.05 versus GFP or *β*-Gal.

Because ROS accumulation at the end of ischemia and during reperfusion exacerbates cardiomyocyte injury [23, 24] and PKC*ε* diminishing these ROS levels is a critical step in preconditioning [3], thus, we tested the role of IEX-1 in H/R-induced ROS accumulation. Neonatal rat cardiomyocytes were infected with 10 MOI of adenovirus and subjected to hypoxia for 4 h, then submitted to chemiluminescence or fluorescence analysis for intracellular ROS accumulation. Intracellular ROS was significantly reduced in cells overexpressing IEX-1 (~74% reduction in chemiluminescence, Figure 6(a), and ~69% reduction in DCF fluorescence, Figure 6(b)).

During H/R, mitochondrion seems to be the main source of ROS in cardiomyocytes [25]. After 4 h hypoxia, cardiomyocytes were incubated with MitoTracker Red CM-H2XRos to probe the mitochondrial ROS (Supplemental Figure 3). Confocal microscopy revealed that neonatal rat cardiomyocytes undergoing H/R displayed a 5.5-fold increase in fluorescence intensity, which was significantly reduced in cells receiving Ad-IEX-1 (Figure 6(c)), indicating that IEX-1 is able to decrease mitochondrial ROS accumulation.

### 3.5. IEX-1 Reduces H/R-Induced Cardiomyocyte Injury

**Figure 7:**
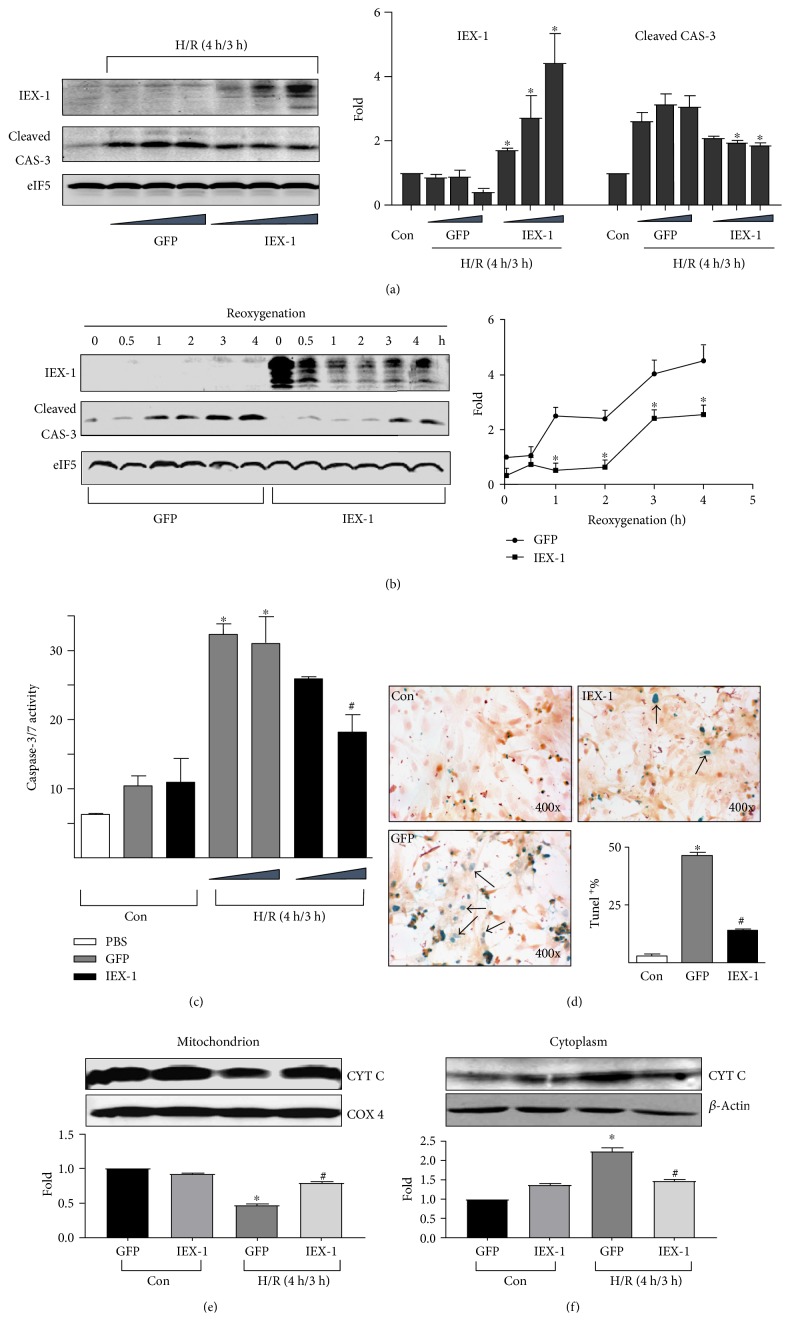
IEX-1 overexpression attenuated H/R-induced cardiomyocyte injury. (a) Cardiomyocytes were infected with Ad-GFP or Ad-IEX-1 (5, 10, and 20 MOI), then underwent H/R. Results are from one representative experiment of three. (b) Cardiomyocytes were infected with 10 MOI adenoviruses, then underwent hypoxia (4 h) and reoxygenation for the indicated time. (c) Cardiomyocytes together with the medium were collected after H/R, total caspase-3/7 activity was detected. Triangle represents adenovirus infection of 5 and 10 MOI. (d) Cardiomyocytes underwent H/R, and TUNEL staining was performed to assess cell apoptosis. Arrows show TUNEL-positive cells, and the ratio of TUNEL-positive cells was calculated. Results are from one representative experiment of four. (e) Mitochondrial and (f) cytoplasmic fractions were isolated from cardiomyocytes, and proteins were probed with an anti-cytochrome c antibody. eIF5, COX4, or *β*-actin expression was a control for protein loading. Each column represents results from at least 3 independent experiments. ^∗^*P* < 0.05 versus control (Con or corresponding time points), ^#^*P* < 0.05 versus the corresponding GFP group.

**Figure 8:**
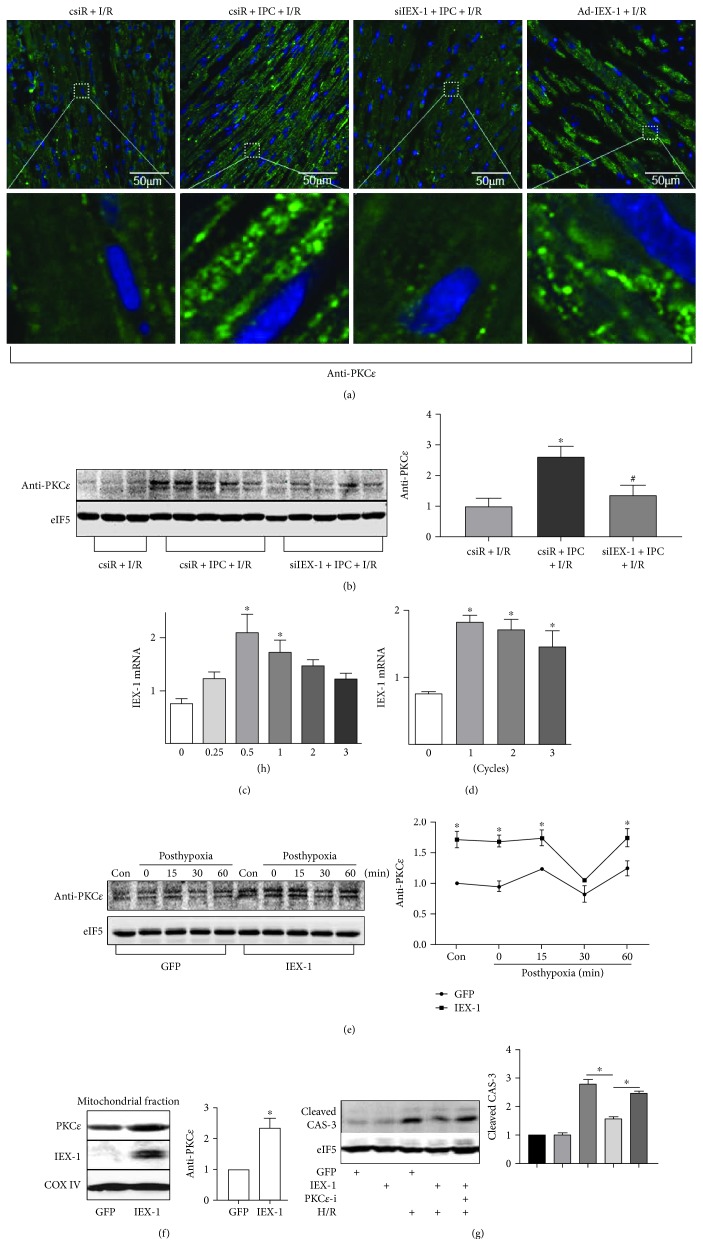
IEX-1 promotes PKC*ε* phosphorylation. (a) Rat hearts were locally delivered with siRNA or Ad-IEX-1, then underwent I/R (30 min/24 h) or IPC prior to I/R as indicated. Particle location of PKC*ε* was identified by immunofluorescence assay with confocal microscopy; (b) the same heart samples were homogenated for Western blot analysis of phosphor-PKC*ε*. (c) Time course of IEX-1 mRNA expression in neonatal rat cardiomyocytes after hypoxia preconditioning (HPC, 1 cycle of short H/R, 10 min each). (d) Effect of increasing HPC cycles on IEX-1 mRNA expression. (e) Neonatal rat cardiomyocytes were infected with adenovirus at 10 MOI for 36 h, then underwent hypoxia (4 h) and reoxygenation for the indicated time. Phosphor-PKC*ε* were detected. (f) Cardiomyocytes were overexpressed with GFP or IEX-1. Mitochondrial particles were isolated and lysed for Western blot. (g) Cardiomyocytes were overexpressed with GFP or IEX-1 or treated with PKC*ε* inhibitor (PKC*ε*V1–2, 20 *μ*M) and then subjected to H/R. ^∗^*P* < 0.05 versus the csiR or 0 group or corresponding time points, ^#^*P* < 0.05 versus csiR + IPC + I/R.

The increased ROS is a primary stimulus for the mitochondrial death pathway. Then, we used an in vitro H/R model to study the effects of IEX-1 on H/R-induced cardiomyocyte death. Neonatal cardiomyocytes were infected with Ad-GFP or Ad-IEX-1 at 5, 10, or 20 MOI. After H/R insult, Ad-IEX-1-infected cells showed less morphological changes than those infected with Ad-GFP (Supplemental Figure 4A). Overexpression of IEX-1, but not GFP, greatly reduced LDH release in conditioned media (Supplemental Figure 4B). As well, caspase-3 cleavage, an indication of apoptosis, reduced in cells overexpressing IEX-1 (Figure 7(a)). After H/R, caspase-3 cleavage increased as reoxygenation time was prolonged in both Ad-GFP- and Ad-IEX-1-infected cells. However, the cleaved caspase-3 was less in cells overexpressing IEX-1 at all time points (Figure 7(b)). Concurrently, caspase-3/7 activity attenuated in cells infected with Ad-IEX-1, compared with those with Ad-GFP (Figure 7(c)). Under H/R, the percentage of TUNEL-positive (apoptotic) cells was also reduced by IEX-1 (Figure 8(d)). Moreover, there was a decrease in mitochondrial cytochrome c leakage to cytoplasm in IEX-1 overexpressed cells (Figures 7(e) and 7(f)). Together, these data indicate that IEX-1 overexpression imposes protective effects on neonatal rat cardiomyocytes under H/R.

### 3.6. IEX-1 Promotes PKC*ε* Phosphorylation and Mitochondrial Translocation

Then, we found clear particle translocation of PKC*ε* in cardiac cells subjected to IPC prior to I/R, which was also observed similarly in the IEX-1 overexpressed heart; in contrast to these, particle translocation of PKC*ε* was significantly inhibited when siIEX-1 was administered to knock down heart IEX-1 (Figure 8(a)). Then, we further detected phosphor-PKC*ε* in hearts after IPC + I/R; of note, the increased level of phosphor-PKC*ε* by IPC was abolished by IEX-1 knocking down (Figure 8(b)).

Multiple studies have demonstrated that activation of protein kinase C*ε* (PKC*ε*) is critical for the protective phenotype of IPC against myocardial I/R injury [5]. Thus, we confirmed these results in cultured cardiomyocytes. First, in vitro IPC, termed hypoxia preconditioning (HPC), induced by short-term hypoxia and reoxygenation similarly increased IEX-1 expression at both the mRNA level (Figure 8(c, d)) and the protein level (Supplemental Figure 5A and B). Thereafter, we overexpressed IEX-1 in cultured cardiomyocytes, and phosphor-PKC*ε* was increased in cells receiving Ad-IEX-1 at both normal culture and post-H/R (Figure 8(e)). Furthermore, Western blot showed that IEX-1 overexpression increased the PKC*ε* protein level in the mitochondrial fraction (Figure 8(f)). And PKC*ε* inhibitor abolished the protective effect of IEX-1 on H/R-induced caspase-3 activation (Figure 8(g)).

## 4. Discussion

This study shows that IEX-1 is pivotal in IPC-induced cardioprotection against I/R-induced injury. We found that IPC, commonly used in coronary intervention and coronary artery bypass grafting [26, 27], could increase IEX-1 expression in ischemic hearts in vivo. When IEX-1 expression was inhibited by siRNA, the protective effects imposed by IPC were lessened. Using gain-of-function approaches, we demonstrated that hearts with exogenously expressed IEX-1 enhanced their tolerance to I/R. In the complementary experiments, siRNA knocking down the endogenous IEX-1 exacerbated the cardiac injury. The molecular basis underlying such a protective effect involves, but not limits to, the promotion of PKC*ε* phosphorylation and attenuation of H/R-induced ROS accumulation and cell death.

Reversible myocardial I/R during human cardiac surgery or mouse IPC is associated with an immediate genomic response [28, 29]. IPC induces the expression of proto-oncogenes or immediate early genes such as *c-Fos*, *c-Myc*, *c-Jun*, and *Egr-1* in isolated rat hearts, which is associated with improved ventricular function and reduced infarct size [7–9]. Our results show that *Iex-1* mRNA was rapidly induced by IPC, suggesting that increased IEX-1 expression would contribute to the protective effects of IPC. The thesis was supported by the fact that siRNA knocking down the endogenous IEX-1 exacerbated the cardiac injury and abolished the protective effects induced by IPC, while overexpressing IEX-1 exhibited similar protective effects to IPC. The antiapoptotic effect of IEX-1 on the cardiomyocyte is in line with several previous studies. IEX-1-transgenic mice showed reduced T-cell apoptosis triggered by FAS ligation or T-cell receptor/CD3 complex [30]. IEX-1 overexpression considerably decreased the p73*β*-mediated and staurosporin-induced CHO cell death [12, 16]. However, IEX-1 has also been shown to increase the rate of apoptosis in CHO cells, HaCaT keratinocytes [15], Hela cells [13], or HEK293 cells [14]. The distinct role of IEX-1 in terms of pro- or antiapoptosis may thus depend on cell types, expression level, and kinds of stimulus. Through transcription factors such as NF-*κ*B/REL complexes, p53, SP1, C-MYC, and AP-1, IEX-1 can be induced in a wide variety of cell types by irradiation, viral infection, inflammatory cytokines, chemical carcinogens, growth factors, and hormones (reviewed in 16), and C-MYC, NF-*κ*B, and AP-1 are IPC responsive [31]; these transcriptional factors are likely to be involved in the immediate induction of IEX-1 by IPC.

Ample evidence implicates that mitochondrion-originated ROS, NADH-dependent microsomes, and NOS are very likely to constitute in the initial injury in cardiomyocytes at the end of ischemia and during reperfusion [25, 32–35]. ROS that resulted from stress can cause lipid peroxidation, protein oxidization, and DNA strand breaks, which affect normal cardiac functions. Although ROS is generated during hypoxia [36], a robust burst of ROS occurs after reoxygenation [37]. In the heart, mitochondria are probably the principal source of ROS after reoxygenation and/or reperfusion [25]. The increased ROS in turn is a primary stimulus for MPTP opening, leading to an impaired mitochondrial membrane [38, 39]. Indeed, opening MPTP, releasing cytochrome c from mitochondria, and activating caspase cascade may be a series of events that induce irreversible injury during reperfusion [39–41]. IEX-1 overexpression reduced ROS accumulation, evidenced by decreased luminal-derived chemiluminescence, DCF fluorescence, and Red CM-H2XRos fluorescence, and limited cytochrome c leakage from mitochondria to cytoplasm. Thus, IEX-1 may be involved in maintaining mitochondrial membrane integrity and preventing the mitochondrial death pathway through inhibiting mitochondrial ROS production.

Mitochondria mediate diverse cellular functions including energy generation and ROS production and contribute to signal transduction. Mitochondria are also key regulators of cell viability and play a central role in necrotic and apoptotic cell death pathways induced by cardiac I/R injury. PKC*ε* plays a critical role in cardioprotective signaling pathways that protect the heart from I/R [42]. Emerging evidence suggests that the cardioprotective target of PKC*ε* resides at the mitochondria, including mitoK_ATP_ (mitochondrial ATP-sensitive K^+^ channel) [43], components of the MPTP [44], and components of the electron transport chain [45], and phosphorylation and mitochondrial translocation of PKC*ε* are required for cardioprotection [46]. It has been reported that cytoplasm location, including mitochondrial location, of IEX-1 is critical for its antiapoptotic and antioxidant effects [12]. In the present study, we found that IEX-1 and PKC*ε* colocalized in mitochondrion; IEX-1 expression is necessary for IPC-induced phosphorylation of PKC*ε* both in vivo and in vitro and promotes mitochondrial translocation of PKC*ε*. Because activating PKC*ε* is critical for IPC-induced cardioprotection against I/R, promoting PKC*ε* activation may be the main mechanism for IEX-1-mediating IPC-induced cardioprotection, as PKC*ε* inhibitor blocked the protective effect of IEX-1. However, the mechanisms for IEX-1-promoting PKC*ε* phosphorylation and mitochondrial translocation remain to be elucidated. Of note, the interaction of IEX-1 with PP2A is the central role for IEX-1-modulating ERK and AKT activity [18, 19], which may also be the mechanism for IEX-1-promoting PKC*ε* activity. On the other side, the potential effects of IEX-1 on ERK and AKT activity may also contribute to the cardioprotection, as the AKT-ERK-NO pathway is another important mechanism for IPC-induced cardioprotection [47].

In summary, we demonstrate that IEX-1 is critical in preventing oxidative stress-induced cardiac injury and mediates IPC-induced cardioprotection in rats. In addition to this newly found antioxidative effect, IEX-1 also exhibits inhibitory effects on hypertension, neointima formation, and cardiac hypertrophy [48–50]. Thereby, IEX-1 would be a cardioprotective gene. The identification of an early response gene involved in I/R-induced heart insult may reveal a new therapeutic strategy to explore targets for ischemic heart diseases.

## Supplementary Material

Supplemental figure 1 (A) IEX-1 protein level in rat hearts post-IPC+I/R were detected by IHC, (B) IHC staining intensity was measured. n=5~6, ∗P<0.05 vs sham, #P <0.05 vs I/R. Supplemental figure 2 (A) Ad-GFP and Ad-IEX-1 expression in rat left-ventricle sections. Rat hearts were excised 4 days after adenovirus injection. GFP expression was detected on fluorescence microscopy (top, magnification ×200). IEX-1 expression was detected by immunohistochemical staining with anti-human IEX-1 antibody (bottom, magnification ×200). (B) Rats underwent surgical thoracotomy, then 5 µg scrambled (csiR) or IEX-1siRNA (siIEX-1) with transfection reagent in 250 µl of saline (NS) was directly injected intramyocardially into the left-ventricular muscular wall. 2 days later, Hearts were excised, sectioned, and stained with anti-IEX-1 antibody. Normal IgG served as negative control. Supplemental figure 3 MitoTracker® Red CM-H2XRos probe was used to locate ROS in mitochondria by a dot distribution pattern. Image acquisition was by confocal microscopy. Supplemental figure 4 IEX-1 overexpression attenuated H/R-induced cardiomyocyte injury. Neonatal rat cardiomyocytes were infected with corresponding adenovirus for 36 hr, then underwent hypoxia for 4 hr. (A) Cardiomyocyte morphology after reoxygenation for 4 hr. Results are from one representative experiment of 3 (magnification ×150). (B) LDH release in the cell culture medium after reoxygenation for 4 hr. Triangle represents adenovirus infection at 5, 10, and 20 multiplicities of infection (MOI). Each column represents results of at least 3 independent experiments. ∗P<0.05 vs. control (Ctrl), #P <0.05 vs. corresponding Ad-GFP group. Supplemental figure 5 Neonatal rat cardiomyocytes were subjected to HPC or HPC+H/R. (A) IEX-1 mRNA was analyzed by real-time PCR. (B) IEX-1 protein was detected by western blot. N=3, ∗ P<0.05 vs Con or H/R. Supplemental table 1. IEX-1 overexpression improves cardiac function after acute I/R.

## References

[1] Braunwald E., Kloner R. A. (1985). Myocardial reperfusion: a double-edged sword?. *The Journal of Clinical Investigation*.

[2] Vinten-Johansen J. (1997). Reperfusion injury: idle curiosity or therapeutic vector?. *Journal of Thrombosis and Thrombolysis*.

[3] Halestrap A. P., Clarke S. J., Khaliulin I. (2007). The role of mitochondria in protection of the heart by preconditioning. *Biochimica et Biophysica Acta (BBA) - Bioenergetics*.

[4] Skyschally A., Schulz R., Heusch G. (2008). Pathophysiology of myocardial infarction: protection by ischemic pre- and postconditioning. *Herz*.

[5] Inagaki K., Churchill E., Mochly-Rosen D. (2006). Epsilon protein kinase C as a potential therapeutic target for the ischemic heart. *Cardiovascular Research*.

[6] Das D. K., Maulik N. (2003). Preconditioning potentiates redox signaling and converts death signal into survival signal. *Archives of Biochemistry and Biophysics*.

[7] Aebert H., Cornelius T., Ehr T. (1997). Expression of immediate early genes after cardioplegic arrest and reperfusion. *The Annals of Thoracic Surgery*.

[8] Das D. K., Engelman R. M., Kimura Y. (1993). Molecular adaptation of cellular defences following preconditioning of the heart by repeated ischaemia. *Cardiovascular Research*.

[9] Zimmermann R., Andres J., Brand T. (1995). Cardiac gene expression after brief coronary occlusion. *Zeitschrift für Kardiologie*.

[10] Wu M. X. (2003). Roles of the stress-induced gene IEX-1 in regulation of cell death and oncogenesis. *Apoptosis*.

[11] Garcia J., Ye Y., Arranz V., Letourneux C., Pezeron G., Porteu F. (2002). IEX-1: a new ERK substrate involved in both ERK survival activity and ERK activation. *The EMBO Journal*.

[12] Shen L., Guo J., Santos-Berrios C., Wu M. X. (2006). Distinct domains for anti- and pro-apoptotic activities of IEX-1. *The Journal of Biological Chemistry*.

[13] Arlt A., Grobe O., Sieke A. (2001). Expression of the NF-*κ*B target gene IEX-1 (p22/PRG1) does not prevent cell death but instead triggers apoptosis in Hela cells. *Oncogene*.

[14] Grobe O., Arlt A., Ungefroren H. (2001). Functional disruption of IEX-1 expression by concatemeric hammerhead ribozymes alters growth properties of 293 cells. *FEBS Letters*.

[15] Schilling D., Pittelkow M. R., Kumar R. (2001). IEX-1, an immediate early gene, increases the rate of apoptosis in keratinocytes. *Oncogene*.

[16] Gonzalez S., Perez-Perez M. M., Hernando E., Serrano M., Cordon-Cardo C. (2005). p73β-Mediated apoptosis requires p57^kip2^ induction and IEX-1 inhibition. *Cancer Research*.

[17] Zhou Q., Hahn J. K., Neupane B. (2017). Dysregulated IER3 expression is associated with enhanced apoptosis in titin-based dilated cardiomyopathy. *International Journal of Molecular Sciences*.

[18] Letourneux C., Rocher G., Porteu F. (2006). B56-containing PP2A dephosphorylate ERK and their activity is controlled by the early gene IEX-1 and ERK. *The EMBO Journal*.

[19] Rocher G., Letourneux C., Lenormand P., Porteu F. (2007). Inhibition of B56-containing protein phosphatase 2As by the early response gene IEX-1 leads to control of Akt activity. *The Journal of Biological Chemistry*.

[20] Zhu Y. H., Ma T. M., Wang X. (2005). Gene transfer of heat-shock protein 20 protects against ischemia/reperfusion injury in rat hearts. *Acta Pharmacologica Sinica*.

[21] Jayasankar V., Woo Y. J., Bish L. T. (2004). Inhibition of matrix metalloproteinase activity by TIMP-1 gene transfer effectively treats ischemic cardiomyopathy. *Circulation*.

[22] Rakhit R. D., Mojet M. H., Marber M. S., Duchen M. R. (2001). Mitochondria as targets for nitric oxide-induced protection during simulated ischemia and reoxygenation in isolated neonatal cardiomyocytes. *Circulation*.

[23] Grill H. P., Zweier J. L., Kuppusamy P., Weisfeldt M. L., Flaherty J. T. (1992). Direct measurement of myocardial free radical generation in an in vivo model: effects of postischemic reperfusion and treatment with human recombinant superoxide dismutase. *Journal of the American College of Cardiology*.

[24] Hearse D. J. (1991). Reperfusion-induced injury: a possible role for oxidant stress and its manipulation. *Cardiovascular Drugs and Therapy*.

[25] Ambrosio G., Zweier J. L., Duilio C. (1993). Evidence that mitochondrial respiration is a source of potentially toxic oxygen free radicals in intact rabbit hearts subjected to ischemia and reflow. *The Journal of Biological Chemistry*.

[26] Jenkins D. P., Pugsley W. B., Alkhulaifi A. M., Kemp M., Hooper J., Yellon D. M. (1997). Ischaemic preconditioning reduces troponin T release in patients undergoing coronary artery bypass surgery. *Heart*.

[27] Marber M. S., Latchman D. S., Walker J. M., Yellon D. M. (1993). Cardiac stress protein elevation 24 hours after brief ischemia or heat stress is associated with resistance to myocardial infarction. *Circulation*.

[28] Arab S., Konstantinov I. E., Boscarino C. (2007). Early gene expression profiles during intraoperative myocardial ischemia-reperfusion in cardiac surgery. *The Journal of Thoracic and Cardiovascular Surgery*.

[29] Konstantinov I. E., Arab S., Li J. (2005). The remote ischemic preconditioning stimulus modifies gene expression in mouse myocardium. *The Journal of Thoracic and Cardiovascular Surgery*.

[30] Zhang Y., Schlossman S. F., Edwards R. A., Ou C. N., Gu J., Wu M. X. (2002). Impaired apoptosis, extended duration of immune responses, and a lupus-like autoimmune disease in IEX-1-transgenic mice. *Proceedings of the National Academy of Sciences of the United States of America*.

[31] Jancso G., Lantos J., Borsiczky B., Szanto Z., Roth E. (2004). Dynamism of NF-κB and AP-1 activation in the signal transduction of ischaemic myocardial preconditioning. *European Surgical Research*.

[32] Ferrari R., Ceconi C., Curello S., Alfieri O., Visioli O. (1993). Myocardial damage during ischaemia and reperfusion. *European Heart Journal*.

[33] Mohazzab H. K., Kaminski P. M., Wolin M. S. (1997). Lactate and PO_2_ modulate superoxide anion production in bovine cardiac myocytes: potential role of NADH oxidase. *Circulation*.

[34] Xia Y., Dawson V. L., Dawson T. M., Snyder S. H., Zweier J. L. (1996). Nitric oxide synthase generates superoxide and nitric oxide in arginine-depleted cells leading to peroxynitrite-mediated cellular injury. *Proceedings of the National Academy of Sciences of the United States of America*.

[35] Heusch G., Boengler K., Schulz R. (2008). Cardioprotection: nitric oxide, protein kinases, and mitochondria. *Circulation*.

[36] Becker L. B., vanden Hoek T. L., Shao Z. H., Li C. Q., Schumacker P. T. (1999). Generation of superoxide in cardiomyocytes during ischemia before reperfusion. *The American Journal of Physiology*.

[37] Kevin L. G., Camara A. K., Riess M. L., Novalija E., Stowe D. F. (2003). Ischemic preconditioning alters real-time measure of O_2_ radicals in intact hearts with ischemia and reperfusion. *American Journal of Physiology-Heart and Circulatory Physiology*.

[38] Halestrap A. P., Clarke S. J., Javadov S. A. (2004). Mitochondrial permeability transition pore opening during myocardial reperfusion—a target for cardioprotection. *Cardiovascular Research*.

[39] Hausenloy D. J., Maddock H. L., Baxter G. F., Yellon D. M. (2002). Inhibiting mitochondrial permeability transition pore opening: a new paradigm for myocardial preconditioning?. *Cardiovascular Research*.

[40] Juhaszova M., Zorov D. B., Kim S. H. (2004). Glycogen synthase kinase-3β mediates convergence of protection signaling to inhibit the mitochondrial permeability transition pore. *The Journal of Clinical Investigation*.

[41] Weiss J. N., Korge P., Honda H. M., Ping P. (2003). Role of the mitochondrial permeability transition in myocardial disease. *Circulation Research*.

[42] Budas G. R., Mochly-Rosen D. (2007). Mitochondrial protein kinase Cε (PKCε): emerging role in cardiac protection from ischaemic damage. *Biochemical Society Transactions*.

[43] Jaburek M., Costa A. D., Burton J. R., Costa C. L., Garlid K. D. (2006). Mitochondrial PKCε and mitochondrial ATP-sensitive K^+^ channel copurify and coreconstitute to form a functioning signaling module in proteoliposomes. *Circulation Research*.

[44] Baines C. P., Song C. X., Zheng Y. T. (2003). Protein kinase Cε interacts with and inhibits the permeability transition pore in cardiac mitochondria. *Circulation Research*.

[45] Ogbi M., Johnson J. A. (2006). Protein kinase Cε interacts with cytochrome c oxidase subunit IV and enhances cytochrome c oxidase activity in neonatal cardiac myocyte preconditioning. *The Biochemical Journal*.

[46] Ohnuma Y., Miura T., Miki T. (2002). Opening of mitochondrial K_ATP_ channel occurs downstream of PKC-ε activation in the mechanism of preconditioning. *American Journal of Physiology-Heart and Circulatory Physiology*.

[47] Cohen M. V., Downey J. M. (2015). Signalling pathways and mechanisms of protection in pre- and postconditioning: historical perspective and lessons for the future. *British Journal of Pharmacology*.

[48] De Keulenaer G. W., Wang Y., Feng Y. (2002). Identification of IEX-1 as a biomechanically controlled nuclear factor-κB target gene that inhibits cardiomyocyte hypertrophy. *Circulation Research*.

[49] Schulze P. C., de Keulenaer G. W., Kassik K. A. (2003). Biomechanically induced gene iex-1 inhibits vascular smooth muscle cell proliferation and neointima formation. *Circulation Research*.

[50] Sommer S. L., Berndt T. J., Frank E. (2006). Elevated blood pressure and cardiac hypertrophy after ablation of the gly96/IEX-1 gene. *Journal of Applied Physiology*.

