# Supplementary material for: Immediate Early Response Gene X-1 (IEX-1) Mediates Ischemic Preconditioning-Induced Cardioprotection in Rats
Source: Oxid Med Cell Longev. 2017 Oct 29;2017:6109061. doi: 10.1155/2017/6109061 (PMC5682079; doi:10.1155/2017/6109061)
Supplement: Supplementary file 1 — Supplemental figure 1 (A) IEX-1 protein level in rat hearts post-IPC+I/R were detected by IHC, (B) IHC staining intensity was measured. n=5~6, ∗P<0.05 vs sham, #P <0.05 vs I/R. Supplemental figure 2 (A) Ad-GFP and Ad-IEX-1 expression in rat left-ventricle sections. Rat hearts were excised 4 days after adenovirus injection. GFP expression was detected on fluorescence microscopy (top, magnification ×200). IEX-1 expression was detected by immunohistochemical staining with anti-human IEX-1 antibody (bottom, magnification ×200). (B) Rats underwent surgical thoracotomy, then 5 µg scrambled (csiR) or IEX-1siRNA (siIEX-1) with transfection reagent in 250 µl of saline (NS) was directly injected intramyocardially into the left-ventricular muscular wall. 2 days later, Hearts were excised, sectioned, and stained with anti-IEX-1 antibody. Normal IgG served as negative control. Supplemental figure 3 MitoTracker® Red CM-H2XRos probe was used to locate ROS in mitochondria by a dot distribution pattern. Image acquisition was by confocal microscopy. Supplemental figure 4 IEX-1 overexpression attenuated H/R-induced cardiomyocyte injury. Neonatal rat cardiomyocytes were infected with corresponding adenovirus for 36 hr, then underwent hypoxia for 4 hr. (A) Cardiomyocyte morphology after reoxygenation for 4 hr. Results are from one representative experiment of 3 (magnification ×150). (B) LDH release in the cell culture medium after reoxygenation for 4 hr. Triangle represents adenovirus infection at 5, 10, and 20 multiplicities of infection (MOI). Each column represents results of at least 3 independent experiments. ∗P<0.05 vs. control (Ctrl), #P <0.05 vs. corresponding Ad-GFP group. Supplemental figure 5 Neonatal rat cardiomyocytes were subjected to HPC or HPC+H/R. (A) IEX-1 mRNA was analyzed by real-time PCR. (B) IEX-1 protein was detected by western blot. N=3, ∗ P<0.05 vs Con or H/R. Supplemental table 1. IEX-1 overexpression improves cardiac function after acute I/R. [file 6109061.f1.doc]

**Supplemental figure 1**

**
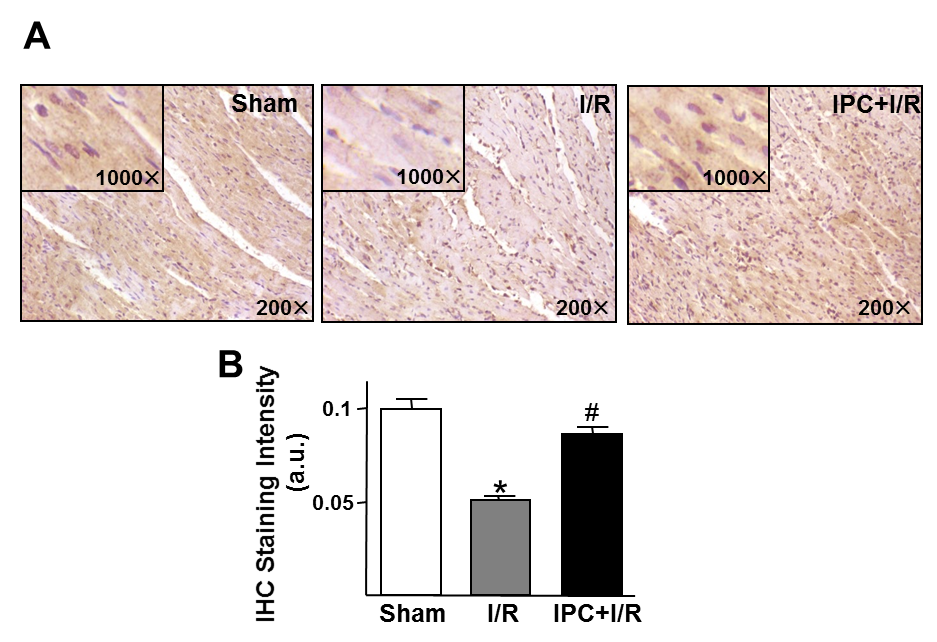
**

**Supplemental figure 1** (A) IEX-1 protein level in rat hearts post-IPC+I/R were detected by IHC, (B) IHC staining intensity was measured. n=5~6, **P*<0.05 vs sham, #*P* <0.05 vs I/R

**Supplemental figure 2**

**A**


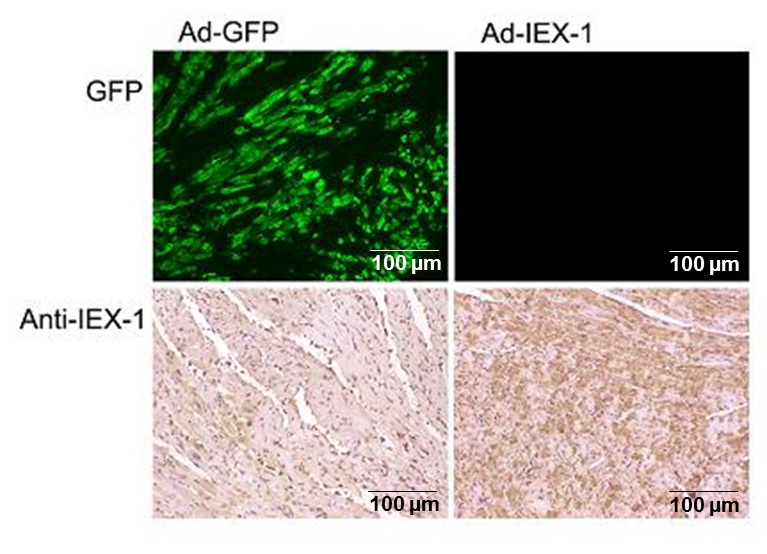


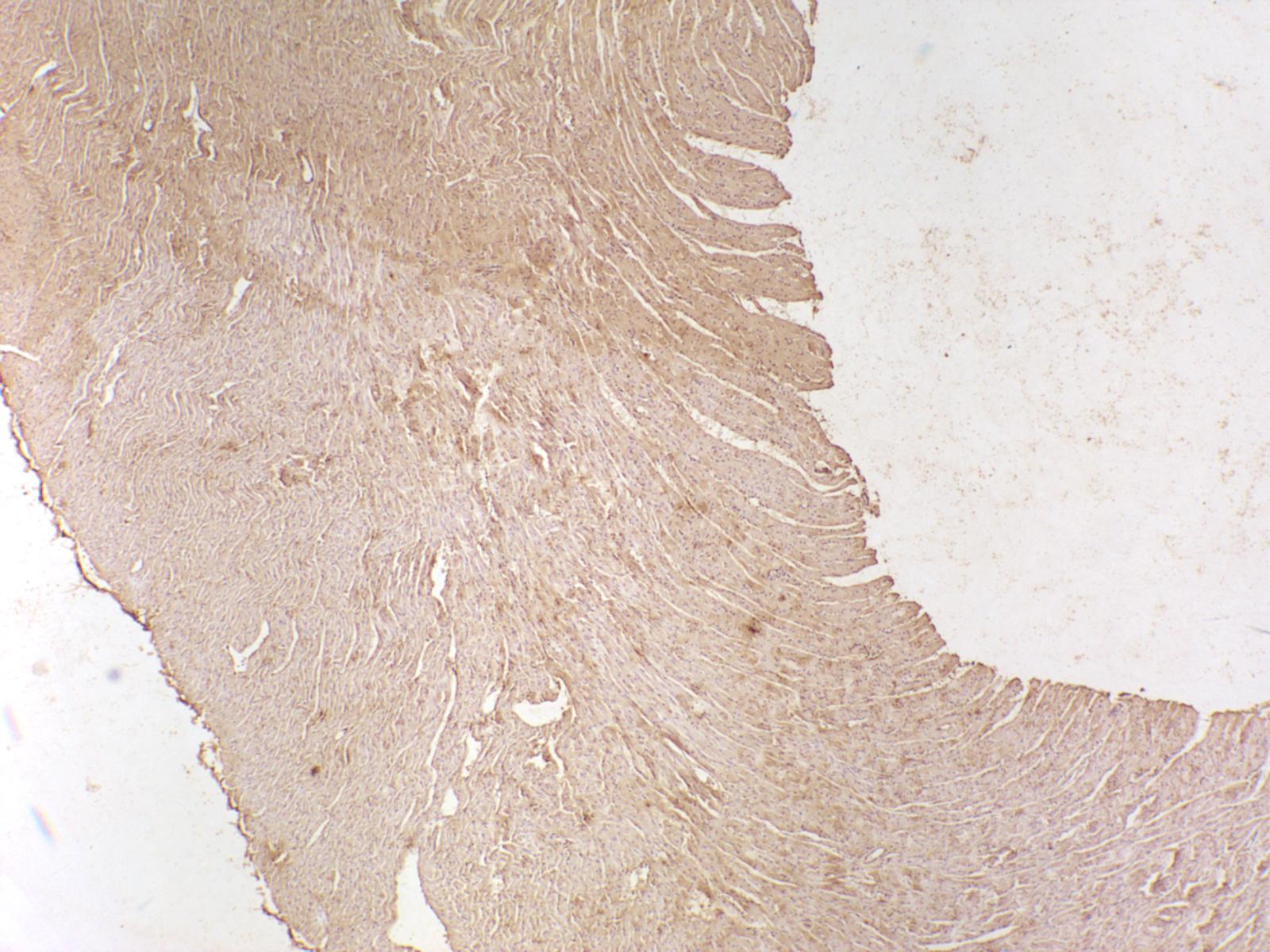

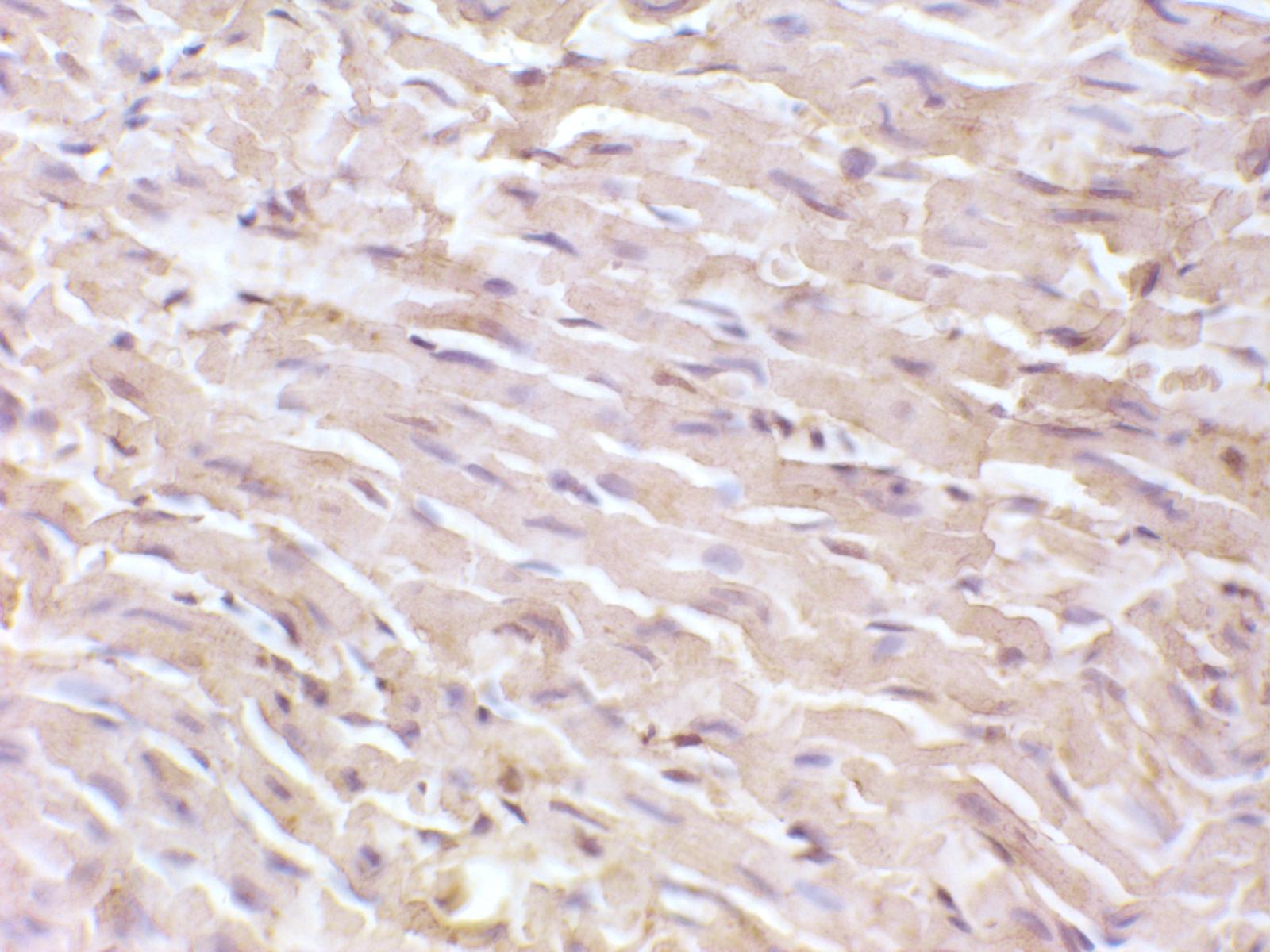

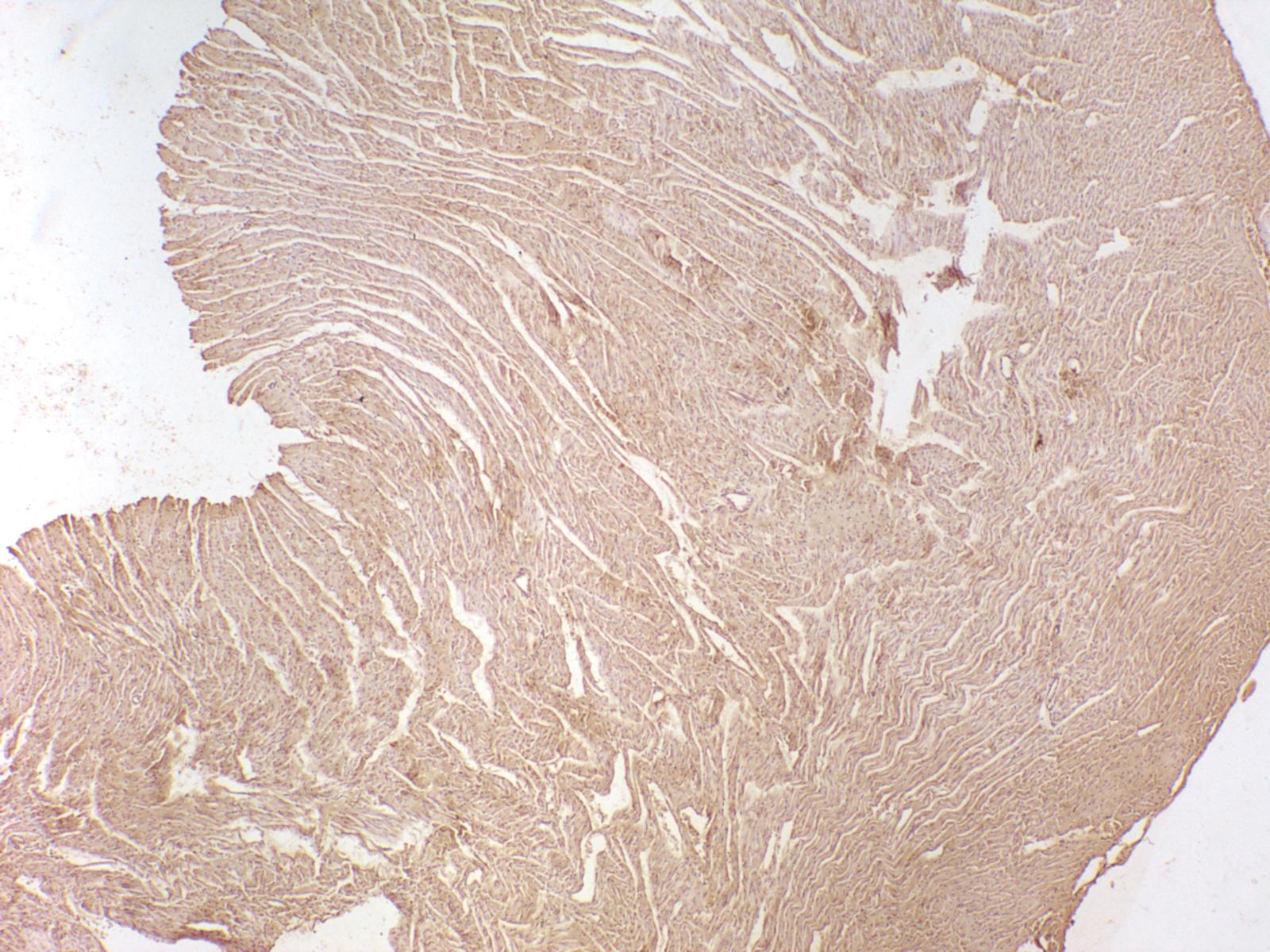

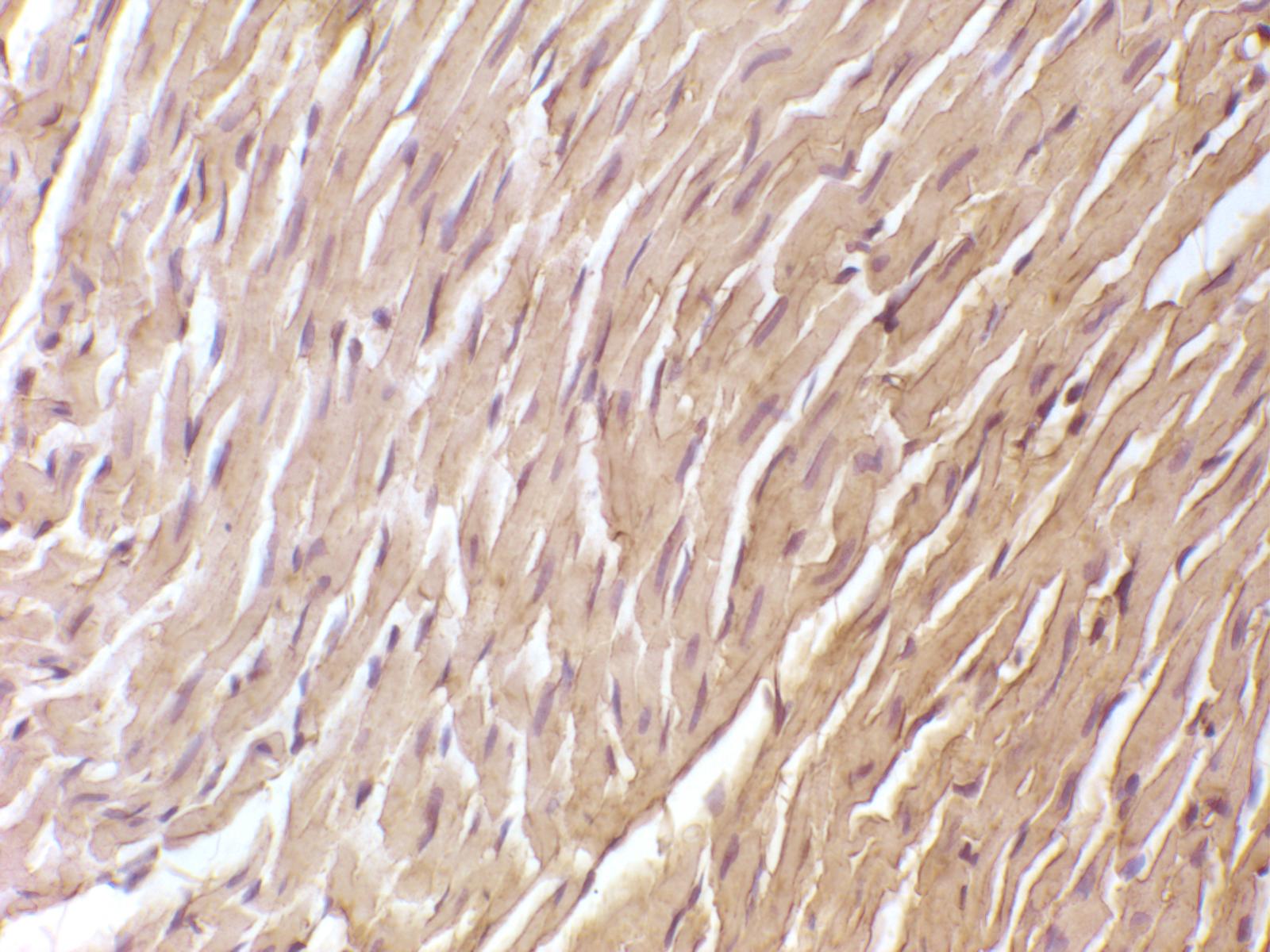

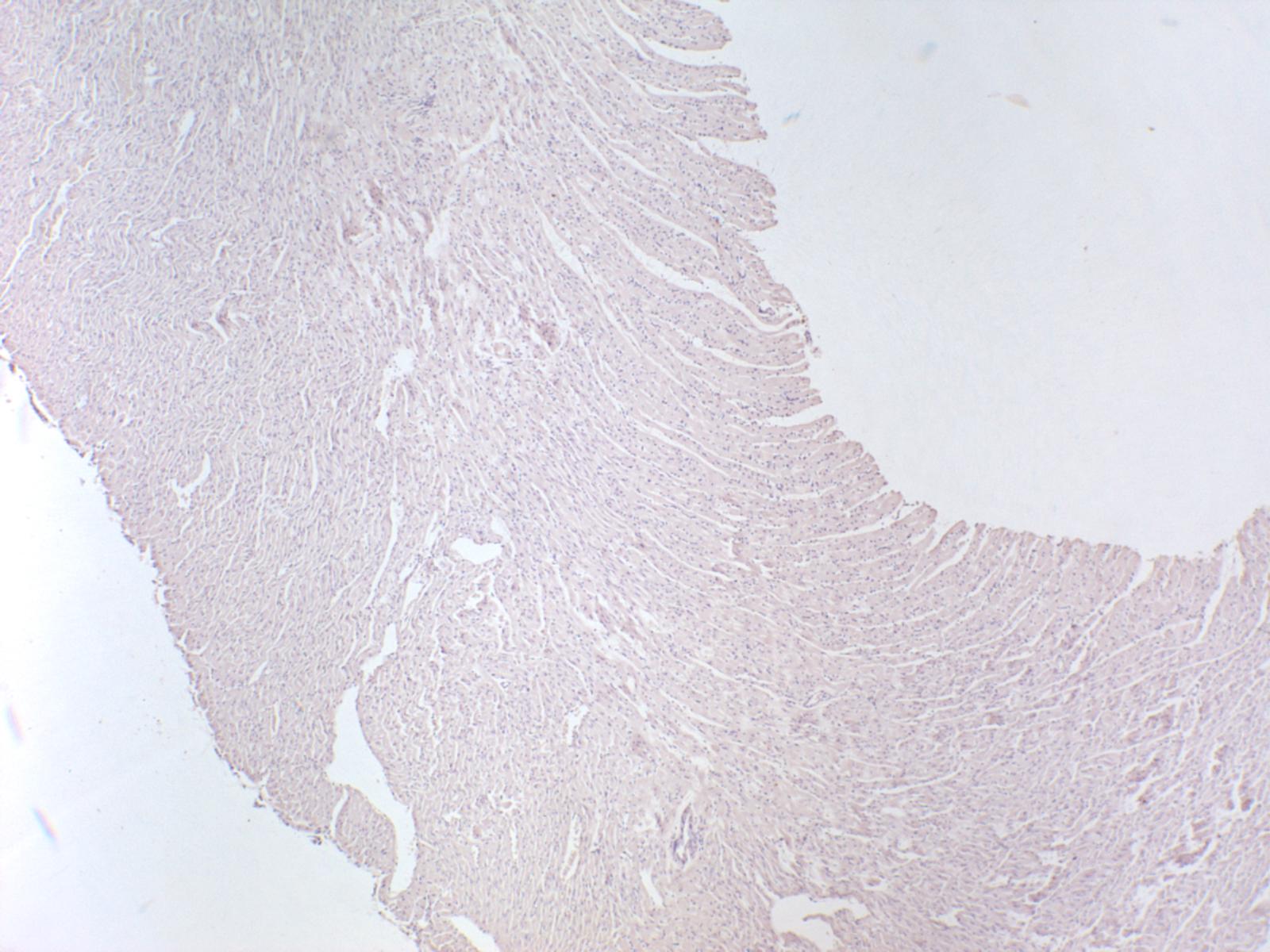

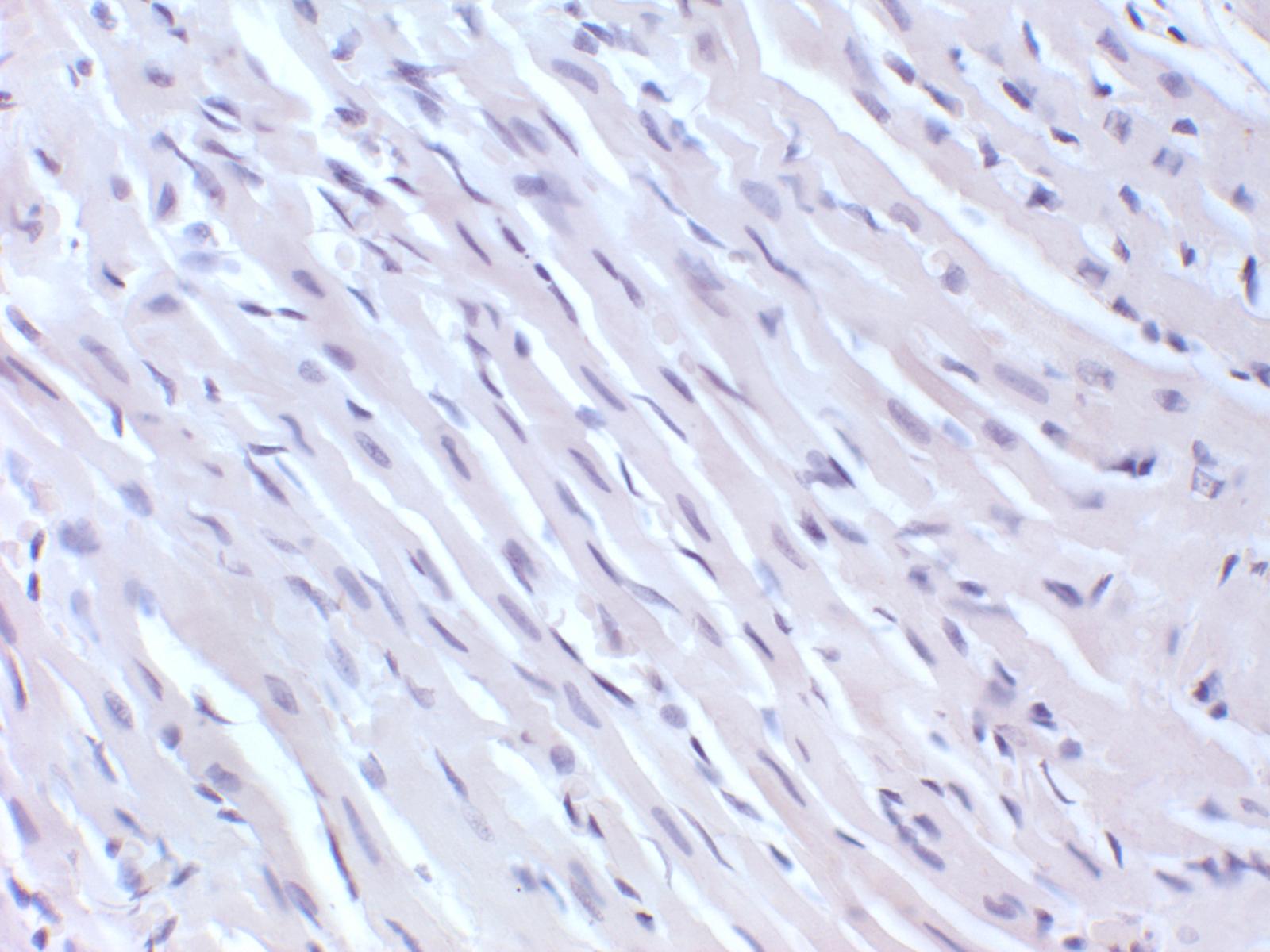


× 800

×40

× 800

×40

× 800

×40

**IgG control**

**siIEX-1**

**csiR**

**B**

**Supplemental figure 2** (A) Ad-GFP and Ad-IEX-1 expression in rat left-ventricle sections. Rat hearts were excised 4 days after adenovirus injection. GFP expression was detected on fluorescence microscopy (top, magnification ×200). IEX-1 expression was detected by immunohistochemical staining with anti-human IEX-1 antibody (bottom, magnification ×200). (B) Rats underwent surgical thoracotomy, then 5 μg scrambled (csiR) or IEX-1siRNA (siIEX-1) with transfection reagent in 250 μl of saline (NS) was directly injected intramyocardially into the left-ventricular muscular wall. 2 days later, Hearts were excised, sectioned, and stained with anti-IEX-1 antibody. Normal IgG served as negative control.

**Supplemental figure 3**

**
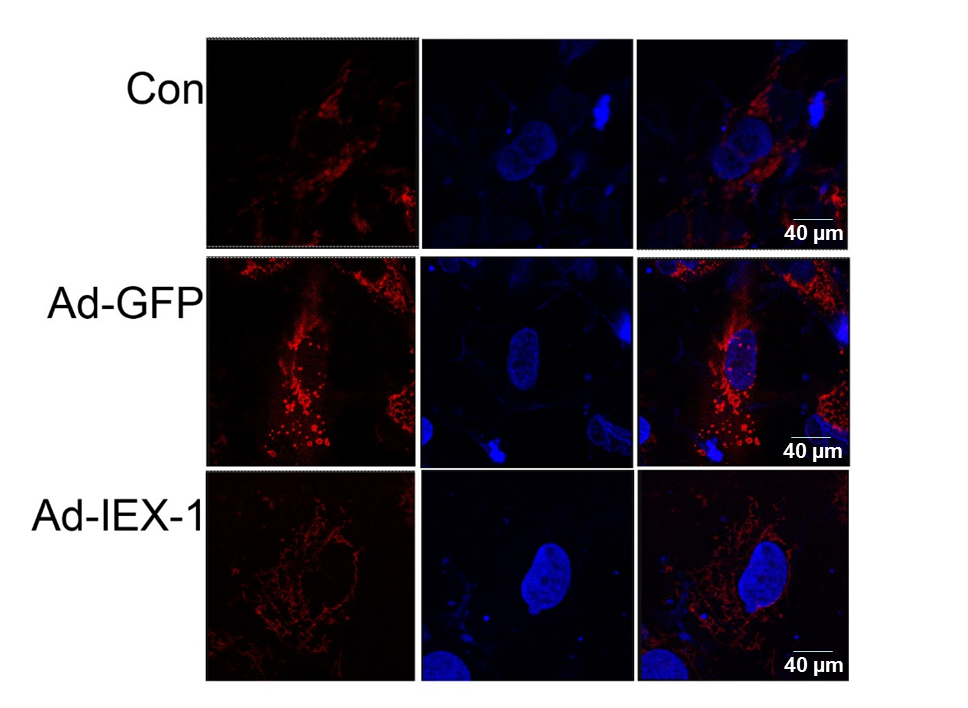
**

**Supplemental figure 3** MitoTracker® Red CM-H2XRos probe was used to locate ROS in mitochondria by a dot distribution pattern. Image acquisition was by confocal microscopy.

**Supplemental figure 4**


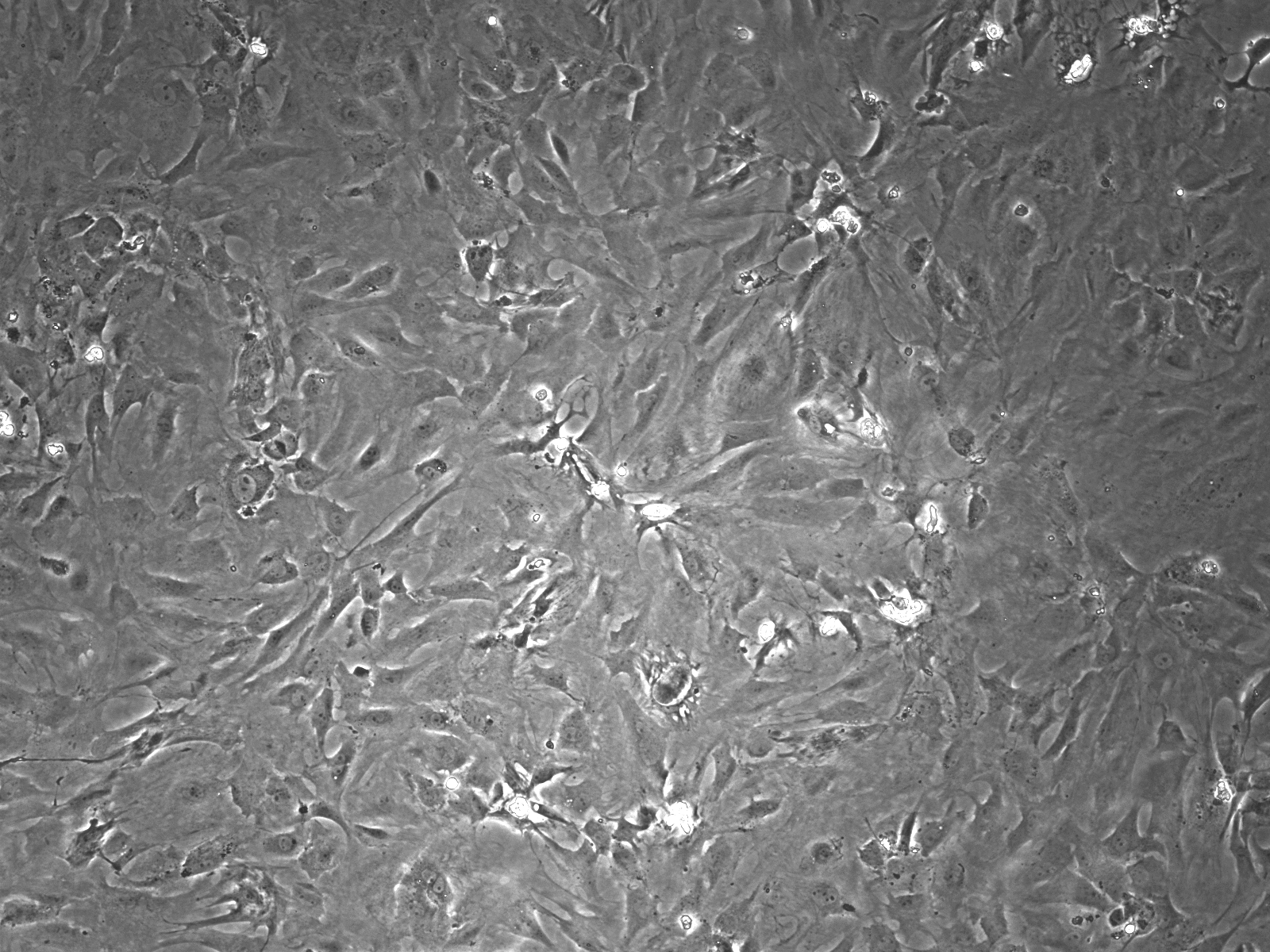

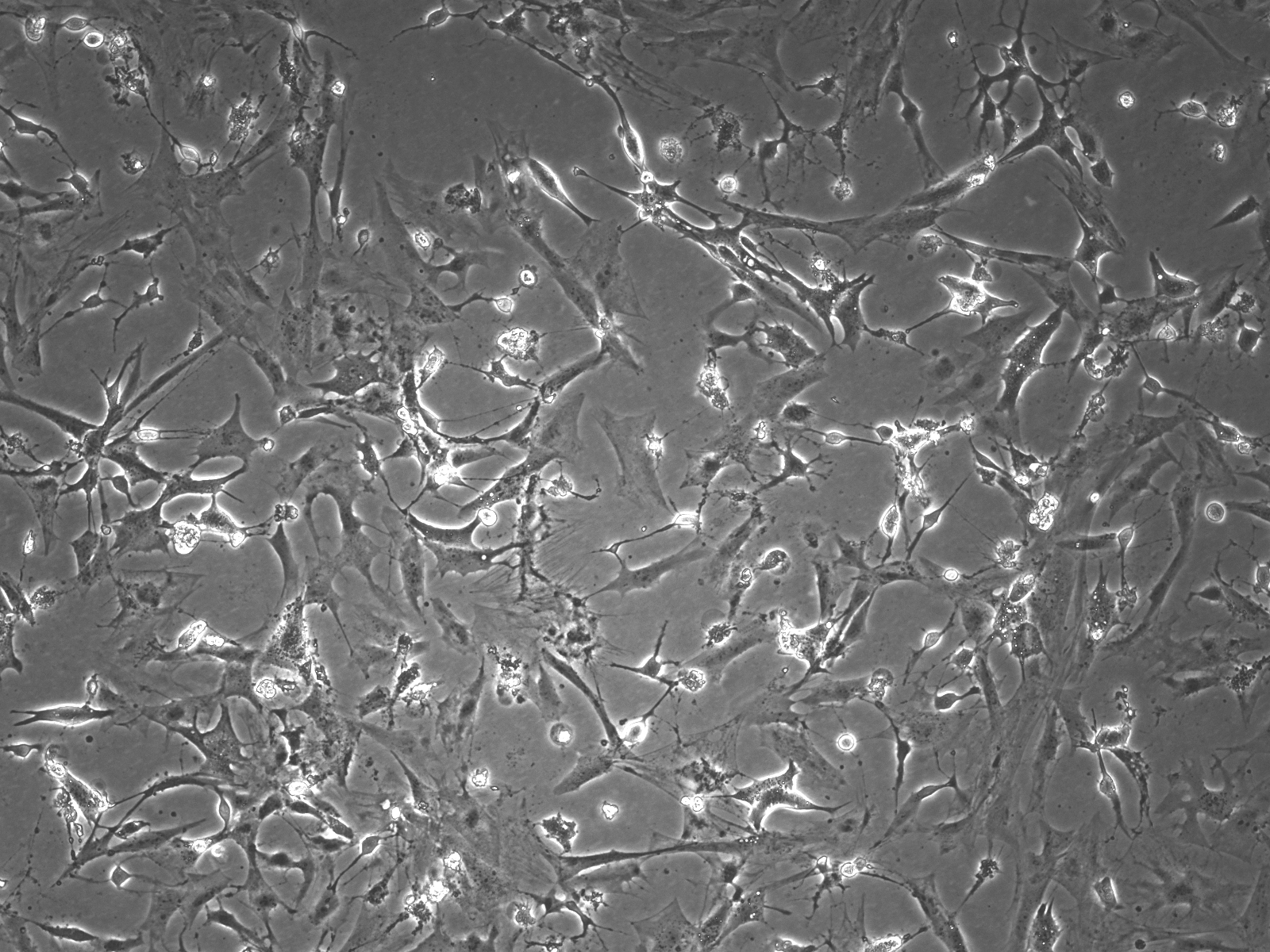

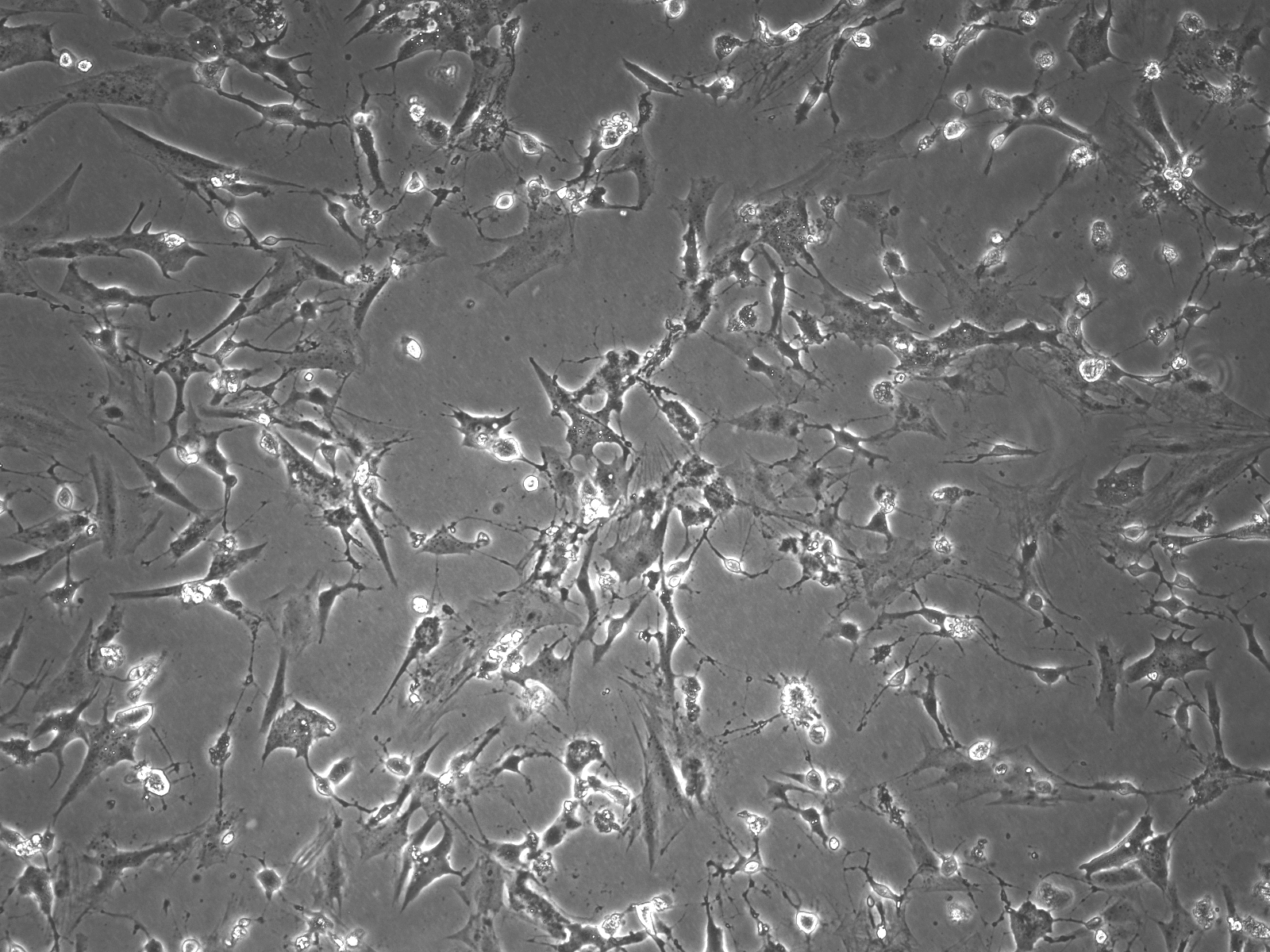

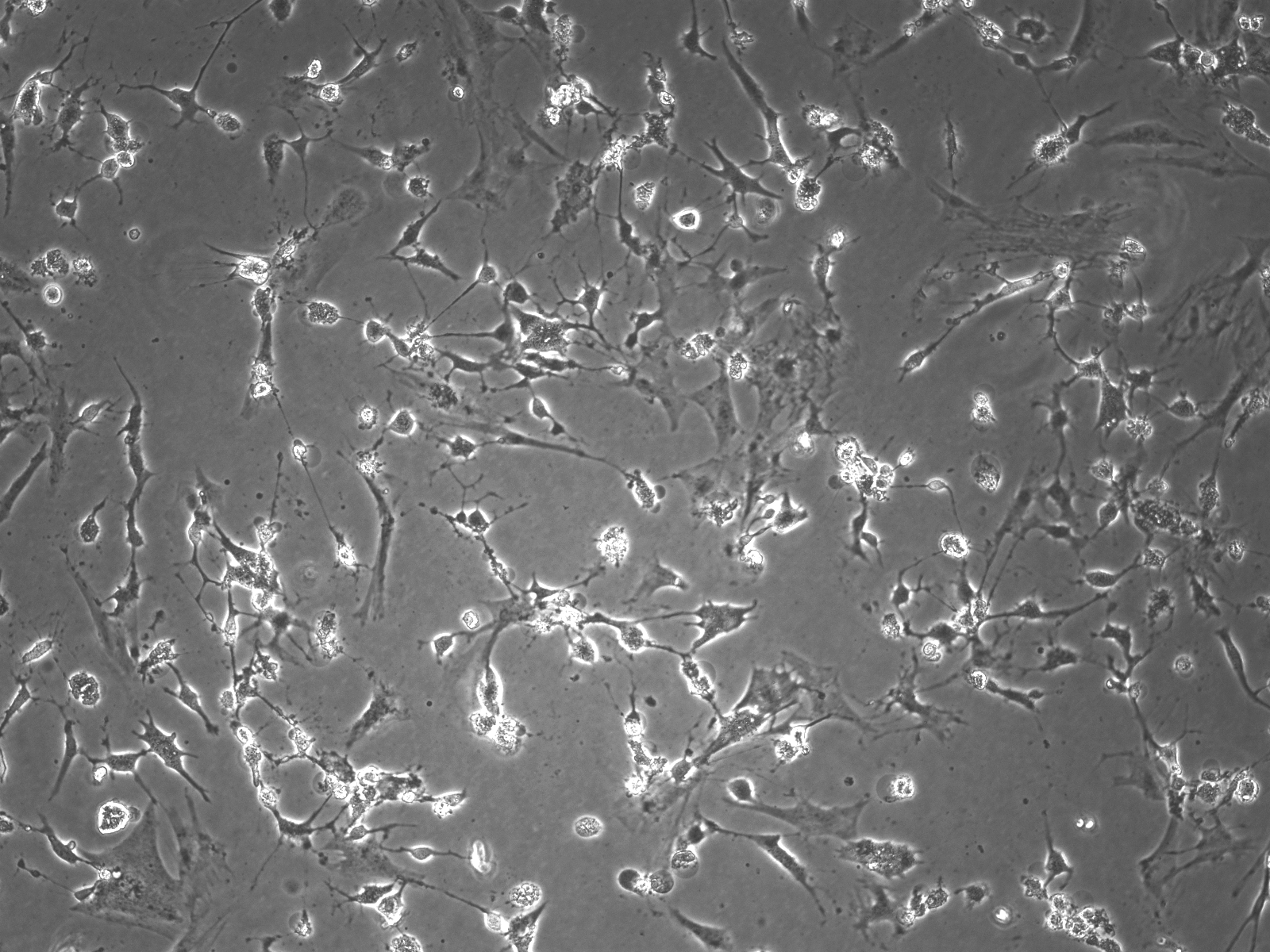

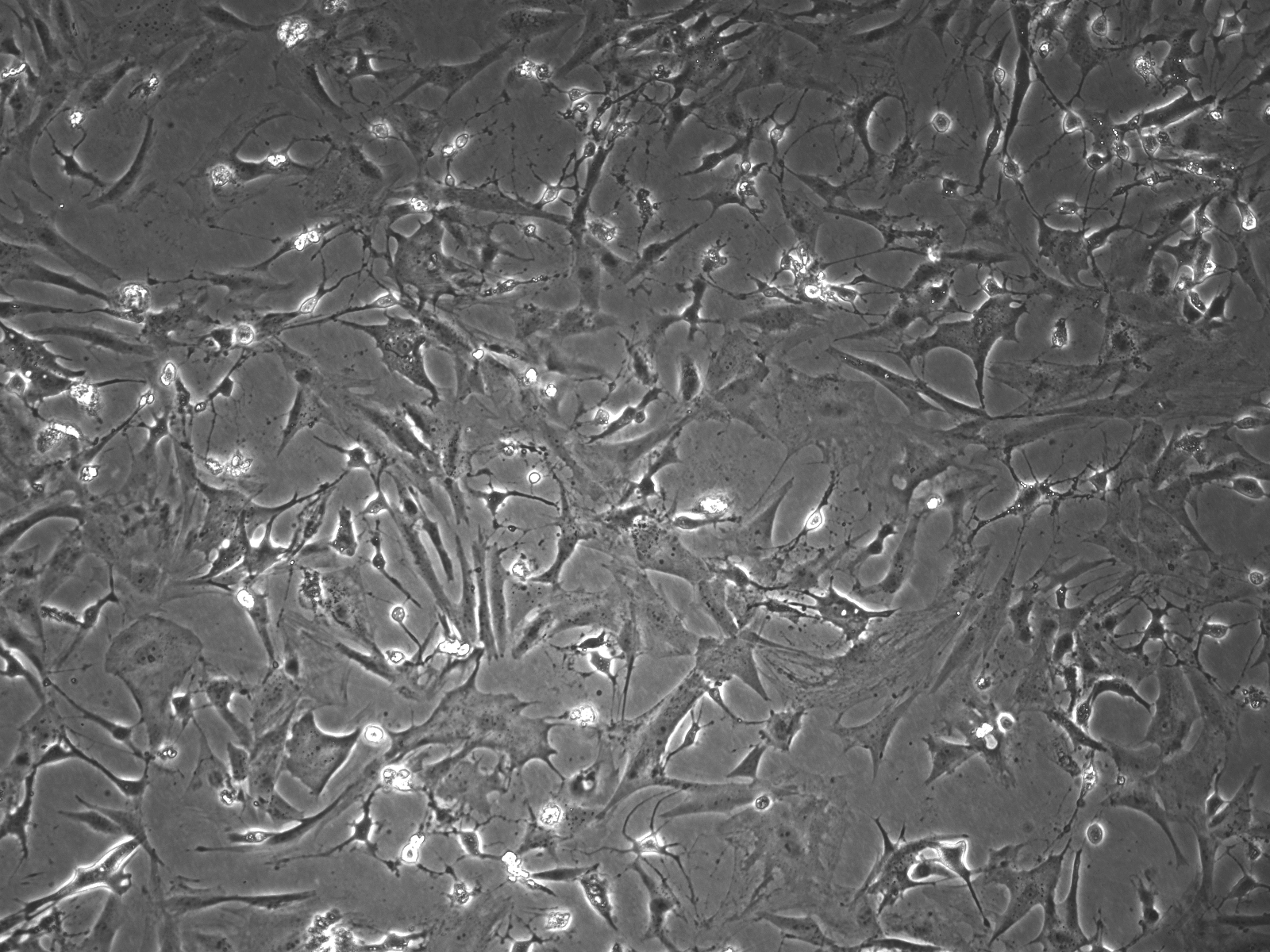

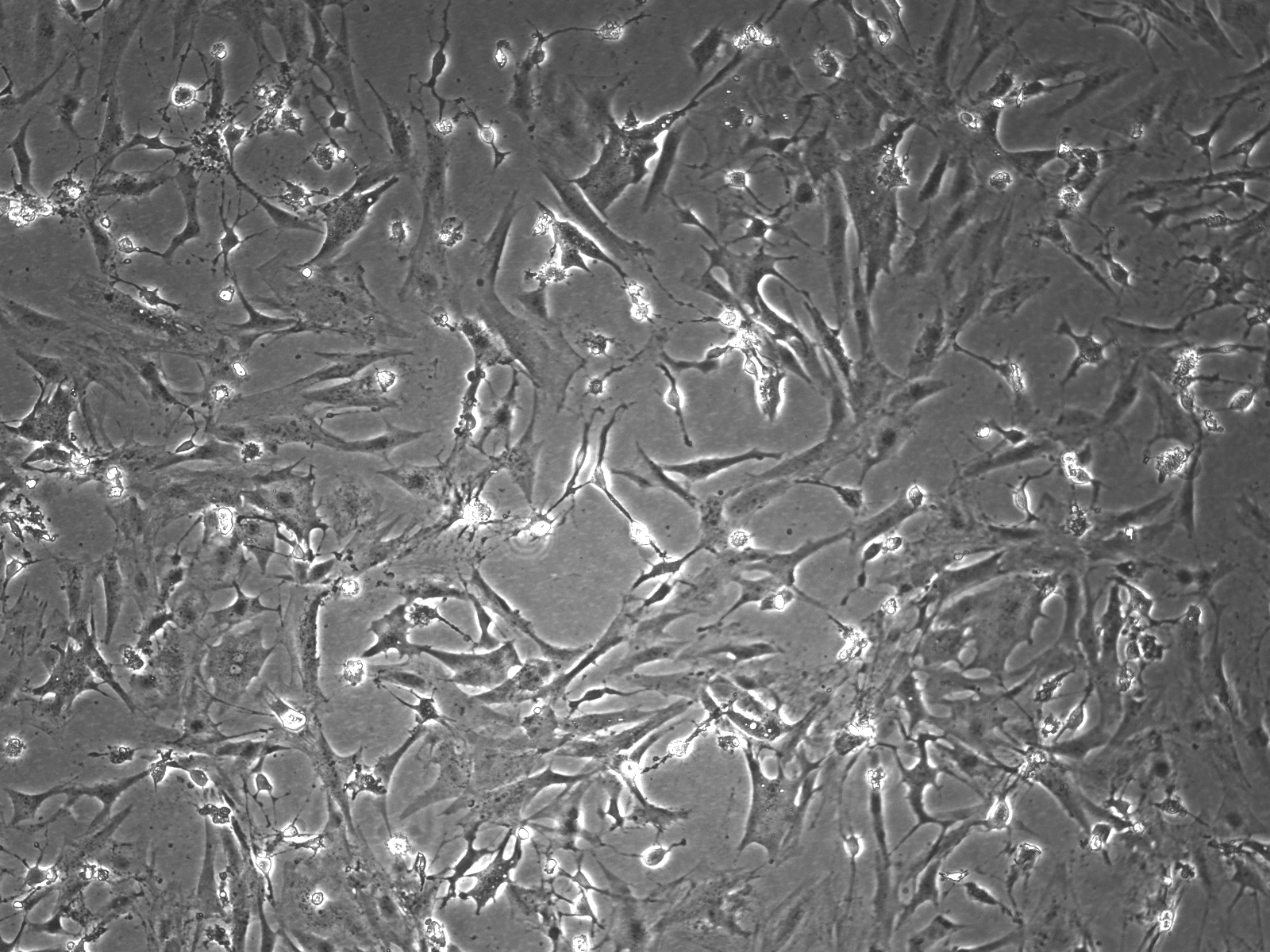

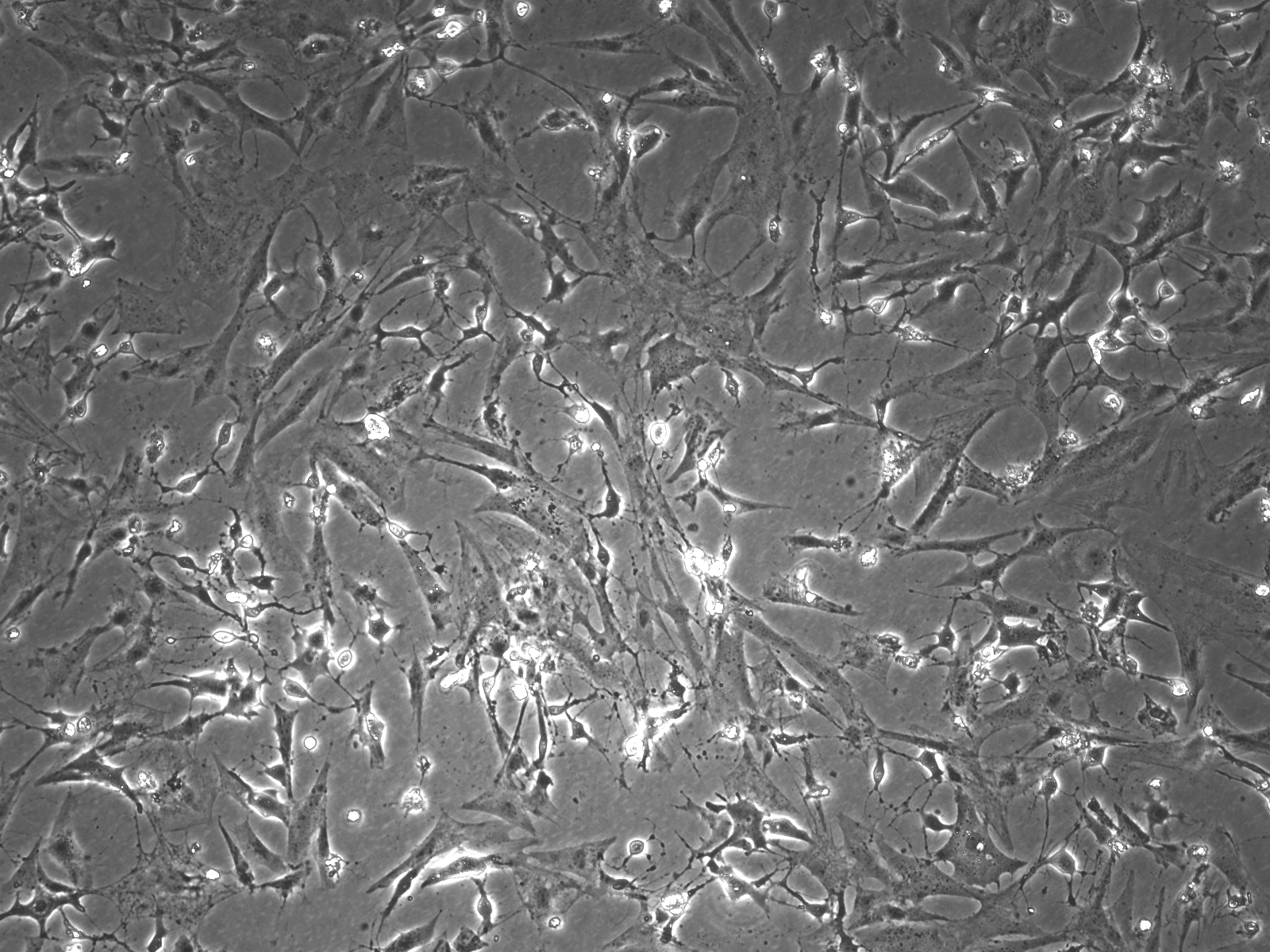


**Con**

**GFP 5**

**GFP 10**

**GFP 20**

**IEX-1 5**

**IEX-1 20**

**IEX-1 10**

**H/R**

**A**


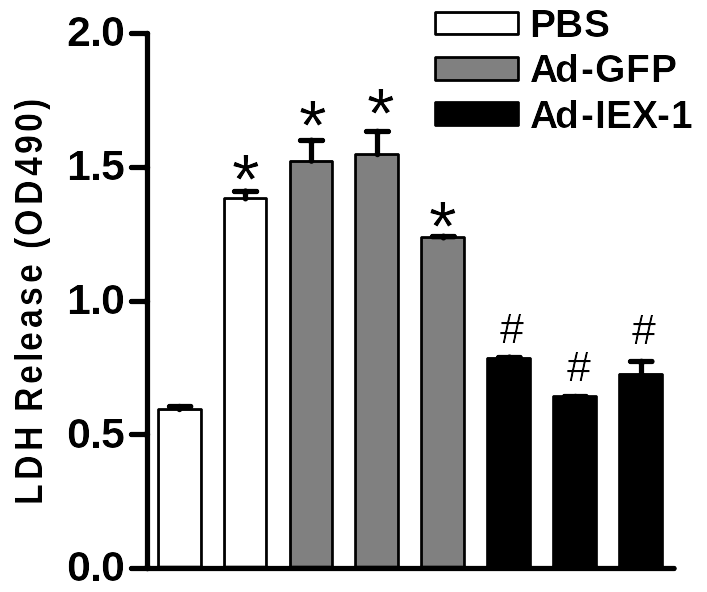


**Ctrl H/R**

**B**

**Supplemental figure 4** IEX-1 overexpression attenuated H/R-induced cardiomyocyte injury. Neonatal rat cardiomyocytes were infected with corresponding adenovirus for 36 hr, then underwent hypoxia for 4 hr. (A) Cardiomyocyte morphology after reoxygenation for 4 hr. Results are from one representative experiment of 3 (magnification ×150). (B) LDH release in the cell culture medium after reoxygenation for 4 hr. Triangle represents adenovirus infection at 5, 10, and 20 multiplicities of infection (MOI). Each column represents results of at least 3 independent experiments. **P*<0.05 *vs.* control (Ctrl), #*P* <0.05 *vs.* corresponding Ad-GFP group.

**Supplemental figure 5**


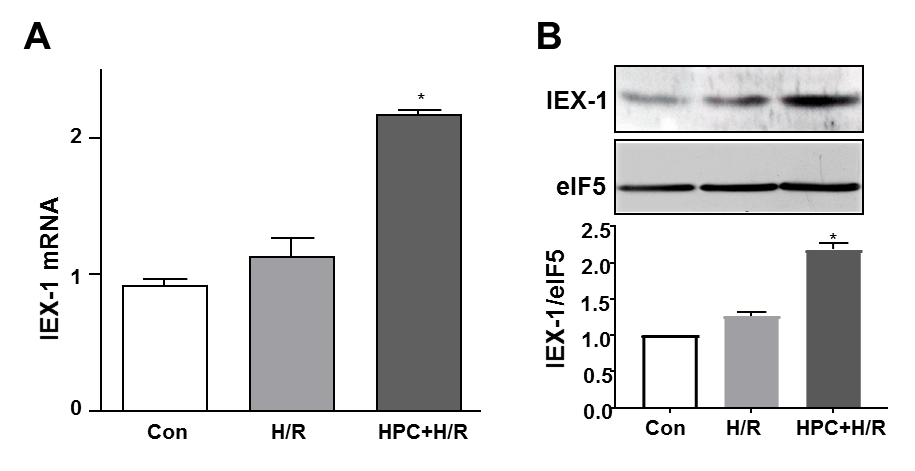


**Supplemental figure 5** Neonatal rat cardiomyocytes were subjected to HPC or HPC+H/R. (A) IEX-1 mRNA was analyzed by real-time PCR. (B) IEX-1 protein was detected by western blot. N=3, * *P*<0.05 vs Con or H/R.

**Supplemental t**able 1. IEX-1 overexpression improves cardiac function after acute I/R

| Groups | Sham (n=8) | NS (n=6) | Ad-GFP (n=6) | Ad-IEX-1 (n=8) |
| --- | --- | --- | --- | --- |
| HR | 415.0± 6.5 | 396.8±12.6 | 391.2±23.8 | 417.3±5.6 |
| SBP | 136.5±2.1 | 112.1±3.2 * | 114.0±1.6* | 118.8±2.4* |
| DBP | 87.5±2.6 | 71.5±3.5* | 66.7±5.2* | 79.5±2.5# |
| MAP | 103.9±2.0 | 85.0±3.0* | 82.5±3.8* | 92.6±2.3 *# |
| LVSP | 157.0±1.5 | 122.3±2.9* | 124.2±2.7* | 132.8±3.6*# |
| +dp/dt_max_ | 8001.1±300.6 | 5816.4±275.7* | 6171.6±365.3* | 6946.5±433.9*# |
| -dp/dt_max_ | -6644.1±187.4 | -4665.5±139.9* | -5111.7±277.8* | -6047.0±345.8# |

HR, heart rate; SBP, systolic blood pressure; DBP, diastolic blood pressure; MAP, mean arterial pressure; LVSP, left ventricular systolic pressure; +dp/dt_max_, maximal rates of pressure increase; -dp/dt_max_, maximal rates of pressure decrease; NS, saline. Data are mean±SEM, **P*<0.05 vs sham, #*P* <0.05 vs NS or Ad-GFP treatment.

**Detailed Materials and Methods**

**Echocardiography**Rats were anesthetized with tribromoethanol and analyzed for anatomy and function on a Vivid 7 Dimension cardiovascular ultrasound system with a 12-MHz microprobe (GE Healthcare). Echocardiographic measurements were taken on M-mode in triplicate from more than 6 separate rats per group.

**Measurement of Infarction**

Myocardium infarct size after acute I/R was determined as described.^1^ Briefly, at the end of the infarction protocol, the ligature around the left anterior descending artery was retightened, and 2 mL of 1% Evans blue dye was injected as a bolus into the LV cavity. Then the heart was excised and cut from apex to base into 6~7 transverse slices of 1-mm thick and incubated in 1% triphenyl tetrazolium chloride solution (TTC) in isotonic pH 7.4 phosphate buffer at 37 °C for 20 min. Viable tissue (red-stained by TTC) was distinguished easily from the infarcted regions (pale or unstained by TTC) and the risk area (unstained by Evans blue). The total slice area (LV), the infarction area (MI), and the area at risk (AAR) of each slice were determined by computer-assisted planimetry (Leica Qwin image analysis software; Leica, Cambridge, UK). During planimetry, the operator was blinded to the type of animal. The ratios of AAR to LV, MI to LV, and MI to AAR were calculated.

**LDH and CK Analysis**

After I/R (30 minutes/24 hours), rat whole blood was collected and centrifugated at 3000 rpm. Serum lactate dehydrogenase (LDH) and creatine kinase (CK) activity was measured on a Hitachi 7170 analyzer with a kinetic enzymatic method to assess heart tissue necrosis.

**Apoptosis Analysis**

Cardiomyocyte apoptosis was analyzed with use of the CardioTACS^TM^ In Situ Apoptosis Detection Kit (R&D Systems, Inc., Minneapolis, MN) and Caspase-Glo® 3/7 Assay Kit (Promega Co., WI) according to the manufacturer’s manual.

**Chemiluminescence Analysis**

The generation of hydrogen peroxide in neonatal rat cardiomyocytes was detected by luminol-plus-horseradish peroxide-derived chemiluminescence in a light-tight box with use of a BPCL Ultra-weak luminescence analyzer (Beijing, China).^7^ Following 4 hr of simulated ischemia, neonatal rat cardiomyocytes were reperfused with 95% air and 5% CO_2_ in DMEM (without serum) for 30 min, then with 3 U/ml horseradish peroxide and 10 mg/ml luminal (Sigma, St. Louis, MO) and immediately examined; the sum counts reflect the formation of hydrogen peroxide.

**Measurement of Intracellular and Mitochondrial ROS Accumulation**

Neonatal rat cardiomyocytes were infected with 10 multiplicities of infection of adenovirus for 36 hr, then hypoxia for 4 hr. After hypoxia, cells were incubated with intracellular ROS probe dye 2’, 7’-dichlorofluorescin diacetate (DCFH-DA, 10 μM; Sigma, St. Louis, MO) or mitochondrial ROS probe dye MitoTracker® Red CM-H2XRos (200 nM; Molecular Probes, Eugene, OR) for 30 min. Determination of intracellular and mitochondrial oxidant production were based on the oxidation of DCFH-DA by intracellular ROS or the oxidation of MitoTracker® Red CM-H2XRos by mitochondrial ROS, which resulted in the formation of the fluorescent compound.^8^ Fluorescence was monitored on confocal laser scanning microscopy (Leica, Bannockburn, IL).

**PCR Analysis**

Total RNA from heart tissue or neonatal rat cardiomyocytes was isolated and reverse transcribed by a reverse transcription system (Promega, Madison, WI). One microliter of the reaction mixture was subjected to PCR. The amount of PCR products formed in each cycle was evaluated by SYBR Green I fluorescence. The forward and reverse PCR primers were rat β-actin 5’-GAG ACC TTC AAC ACC CCA GCC-3’ and 5’-TCG GGG CAT CGG AAC CGC TCA-3’; and rat IEX-1 5'-CGT GCG TCC GAA CAC TTC TC-3' and 5'-CCT GCG ACA CAC CTT CTT CAG-3'. All amplification reactions involved use of the Mx3000 Multiplex Quantitative PCR System (Stratagene, La Jolla, CA).

**Western Blot Analysis**

Following treatment, cardiomyocyte cytosolic, mitochondrial or total proteins were collected as previously described by our laboratory.^9^ Proteins were subjected to SDS-PAGE and then transferred to a nitrocellulose membrane. The membranes were incubated successively with 3% bovine serum albumin and different primary antibodies (primary antibodies of IEX-1 [human or rat], SOD-1, SOD-2, cytochrome c and eIF5 purchased from Santa Cruz Biotechnology [Santa Cruz, CA], anti-cleaved-caspase-3 antibody from Cell Signaling Technology [Beverly, MA], then IRDye^TM^ conjugated second antibody [Rockland Inc, Gilbertsville, PA]). Then the immunofluorescence band was detected with use of the Odyssey infrared imaging system (LI-COR Biosciences, Lincoln, NE).

**References**

**1.** Zhu YH, Ma TM, Wang X. Gene transfer of heat-shock protein 20 protects against ischemia/reperfusion injury in rat hearts. *Acta Pharmacol Sin.* 2005;26(10):1193-1200.

**2.** Schultz JE, Rose E, Yao Z, Gross GJ. Evidence for involvement of opioid receptors in ischemic preconditioning in rat hearts. *Am J Physiol.* 1995;268(5 Pt 2):H2157-2161.

**3.** Jayasankar V, Woo YJ, Bish LT, Pirolli TJ, Berry MF, Burdick J, Bhalla RC, Sharma RV, Gardner TJ, Sweeney HL. Inhibition of matrix metalloproteinase activity by TIMP-1 gene transfer effectively treats ischemic cardiomyopathy. *Circulation.* 2004;110(11 Suppl 1):II180-186.

**4.** Wang J, Xu N, Feng X, Hou N, Zhang J, Cheng X, Chen Y, Zhang Y, Yang X. Targeted disruption of Smad4 in cardiomyocytes results in cardiac hypertrophy and heart failure. *Circ Res.* 2005;97(8):821-828.

**5.** Rakhit RD, Mojet MH, Marber MS, Duchen MR. Mitochondria as targets for nitric oxide-induced protection during simulated ischemia and reoxygenation in isolated neonatal cardiomyocytes. *Circulation.* 2001;103(21):2617-2623.

**6.** Uchiyama T, Engelman RM, Maulik N, Das DK. Role of Akt signaling in mitochondrial survival pathway triggered by hypoxic preconditioning. *Circulation.* 2004;109(24):3042-3049.

**7.** Chen M, Li W, Wang N, Zhu Y, Wang X. ROS and NF-kappaB but not LXR mediate IL-1beta signaling for the downregulation of ATP-binding cassette transporter A1. *Am J Physiol Cell Physiol.* 2007;292(4):C1493-1501.

**8.** Zeng X, Dai J, Remick DG, Wang X. Homocysteine mediated expression and secretion of monocyte chemoattractant protein-1 and interleukin-8 in human monocytes. *Circ Res.* 2003;93(4):311-320.

**9.** Wang W, Jia L, Wang T, Sun W, Wu S, Wang X. Endogenous calcitonin gene-related peptide protects human alveolar epithelial cells through protein kinase Cepsilon and heat shock protein. *J Biol Chem.* 2005;280(21):20325-20330.
